# A comparative study of dynamic risk spillovers among financial sectors in China before and after the epidemic

**DOI:** 10.1371/journal.pone.0314071

**Published:** 2024-12-10

**Authors:** Cuicui Liu, HuiZi Ma, Xiangrong Wang, Junfu Cui, Xu Shen

**Affiliations:** 1 College of Mathematics and Systems Science, Shandong University of Science and Technology, Qingdao, Shandong, China; 2 School of Economics (School of Sci-Tech and Finance), Shandong Women’s University, Jinan, Shandong, China; Wenzhou University, CHINA

## Abstract

This paper takes the unexpected event of the new coronavirus as the research background, selects the daily closing price data of the financial sectors (banking, insurance, securities, and multifinance) from 20 June 2017 to 31 December 2023. It then applies the TVP-VAR-DY model to empirically study the risk spillover effect among financial sectors. The study identified three distinct stages: before, during, and after the epidemic. It revealed that the total systematic spillover exhibited an initial increase, followed by a subsequent decrease. Notably, the fluctuation in this phenomenon intensified significantly during the epidemic. The securities sector emerged as the most susceptible to spillover risks from other sectors and, in turn, the most vulnerable to risk contagion from other sectors. Conversely, the banking sector demonstrated relative stability. Furthermore, the multifinance sector is more susceptible to risk contagion from other sectors during the pre-epidemic and mid-epidemic stages. However, following the epidemic, as the economy has not yet fully recovered, the multifinance sector is more likely to experience spillover risks from other sectors, and the insurance sector also primarily acts as a risk spillover. Finally, five different lag orders were selected to test the robustness of the empirical results of the model. The test results demonstrated that the model was valid and the results were feasible.

## 1. Introduction

In recent years, the frequent occurrence of financial events such as the US-China trade war and P2P platform mines has led to the global economy and financial markets being plunged into a world of chaos, with global stock markets dipping, exchange rates depreciating, and economic downward pressure intensifying. This, coupled with the new crown epidemic that began in 2020, has once again fuelled market turmoil. The Chinese government has consistently prioritised the prevention of systemic financial risks. This is evidenced by the explicit listing of the prevention and resolution of major risks as one of the three major battles to be fought at the 18th, 19th and 20th CPC National Congresses. Furthermore, financial regulators have repeatedly emphasised the importance of maintaining the stability of the financial system, stating that "we will resolutely hold the bottom line of not incurring any systemic financial risks". Although the novel coronavirus (COVID-19) pandemic has receded from the public consciousness, its impact on the global financial system remains significant. As the core of the modern economy, maintaining the stable development of finance is an important safeguard and measure for promoting the healthy, stable and high-quality development of the socialist market economy. One of the important features of the financial market is the risk contagion and spillover effect within the entire financial system, and accurately portraying the risk contagion effects between financial sectors is an important prerequisite for preventing and avoiding financial risks. Based on the above background and analysis, how to better measure the systemic risk transmission and spillover structure among financial sectors under emergencies, explore the systemic risk mechanism of the financial system under emergencies, and timely grasp the direction and intensity of risk spillovers from each sector in the financial system, as well as the differential changes of each sector before, during and after emergencies, are of great significance for maintaining market stability, and preventing and resolving financial risks, which has also become one of the important topics discussed by academics and financial regulators.

## 2. Literature review

A large number of scholars have conducted research on this topic, with representative metrics including the financial composite index method, tail metrics (based on incremental conditional insured value ΔCoVaR and marginal expected loss MES), and the network topology method, etc.. All of the aforementioned methods have yielded more effective results in systemic risk metrics.

The first type of method for constructing financial composite indices. One frequently used constructed index is the financial stress index. For example, Illing and Li (2006) constructed the Canadian Financial Stress Index through the data of financial institutions in Canada [1]. Zhang Zongxin (2022) constructed cross-sectoral risk spillover networks based on the financial stress index method to measure systemic financial risk dynamics, and demonstrated the structural differences and systemic importance of the role of multidimensional risk factors in driving systemic financial risk [2]. In addition to the above methods, Acharya et al. (2010) proposed the degree of capital shortfall as a method. The indicator for identifying systemically important financial institutions is the individual financial institutions’ contribution to the financial system as a whole, which is also known as the short-run marginal expected loss (MES) [3]. Subsequently, Brownlees and Engle (2011) improved the MES and constructed the index that can identify the systematic risk index SRISK for long-term expected capital shortfall [4]. Liang Qi (2013) also improved the SRISK method in terms of validity and measurement model, and calculated the degree of capital shortage of 34 listed financial institutions in China [5]. Zhang Jinqing (2021) introduced a quantitative description of the credit overexpansion scenario, corrected the pro- cyclical problem of the SRISK indicator, and measured the size of systemic risk in China’s banking sector based on the correction of this indicator [6]. In conclusion, this type of financial composite index has a commonality, that is, it needs a huge number of indicators to support, how to select the indicators and reflect the weighting between the indicators has become the key point of this kind of methodology. Otherwise, it may lead to the invalidation of the main indicators affecting the systematic risk, which deviates from the actual financial risk, so that the financial system risk measurement model loses its theoretical significance.

The second type is tail metric. Tail risk is an important pathway for systemic risk contagion and spillover when extreme events occur. This class of methods is derived from VaR (Value at Risk). VaR is a measure of the maximum possible loss of a financial asset over a given holding period and at a given confidence level. However, it does not adequately portray a country’s overall financial risk and may underestimate risk spillovers across different financial sectors. Therefore, Adrian and Brunnermeier (2008, 2016) proposed CoVaR(conditional value-at-risk) and ΔCoVaR (incremental conditional value-at-risk) models on the basis of VaR [7], which focus more on the use of tail correlations among financial institutions to measure risk spillovers among individual financial institutions, as well as the marginal contribution of individual financial institutions to the overall systemic risk. Studies have demonstrated that it can better reflect the dynamic changes of systemic risk in the time dimension, and can also more accurately predict and analyse the future. Consequently, it has been widely adopted by scholars. Gao (2011) used the CoVaR model to study the systemic risk contribution of 14 listed commercial banks in China, and concluded that the risk contagion and spillover of joint-stock banks is smaller than that of state-owned banks [8]. Chen Jianqing (2015) constructed static and dynamic CoVaR models to study the systemic financial risk spillover effect between financial industries in China from two perspectives. The findings revealed that there are positivity and asymmetry between financial industries [9]. Furthermore, to address the asymmetry of the financial industry, Chen Guojin (2017) constructed an asymmetric CoVaR model based on the systematic correlation, and measured the systematic correlation between a single bank and the banking system in China, as well as the systematic correlation between any two banks [10]. Li Zheng (2019) used downward and upwardΔCoES indicators to study the asymmetry between financial sectors as well [11]. Additionally, some scholars have concentrated on the comparative analysis of various types of methods. For instance, Yang Zihui et al. (2018) used four types of risk measures, VaR, MSE, CoVaR and ΔCoVaR, to compare and study the systemic financial risk between financial institutions and real estate firms. Additionally, they examined the cross-sectoral contagion of financial risk [12]. Additionally, there are also scholars who combine various types of models to utilise them in conjunction with one another. For example, Yuting Wang (2019) constructed a binary DCC-GARCH-CoVaR model to investigate the risk spillover effects between Internet money funds and commercial banks in China [13]. Li Zhu (2021) explored the risk spillover between Internet finance and traditional finance based on Copula-ARMA-GARCH-CoVaR model [14]. In a recent study, Li Qiang et al. (2022) investigated the offshore and onshore RMB interest rate risk spillover using the MSGARCH-Mixture Copula model [15]. In recent years, a number of scholars have begun to explore the effectiveness of various types of risk measures. For example, Ouyang Zisheng (2023) backtested commonly used systemic risk measures such as MES, CoVaR andΔCoVaR using the unconditional coverage test method. The results demonstrated that these methods were ineffective in measuring risk during the crisis period [16]. In conclusion, the majority of scholars concur with the tail risk model. However, empirical evidence indicates that although the CoVaR model can be used to measure the direction and strength of the tail risk correlation between two financial markets, it is unable to account for the risk spillover relationship between multiple financial markets simultaneously. Furthermore, the effectiveness of the risk measurement during periods of crisis is limited, which needs to be further improved.

The third type is network analysis method. This is a new perspective of research in this field. this type of method is based on the vector autoregressive model (VAR) and variance decomposition. The VAR model can only study the direction of spillovers between different industries, but cannot obtain the specific spillover values. In order to characterise the dynamics of the spillover effect, Diebold & Yilmaz (2014) used the rolling window VAR method for parameter estimation and variance decomposition within the sample [17], which enables the dynamic estimation of total and directed spillover indices. However, the VAR method necessitates the use of a fixed time window, and there is a strong human subjective factor for the selection of the window width. Inaccurate estimation results are likely to occur when the window width is either too short or too long. At the same time, due to the existence of the window width, the dynamic spillovers ultimately obtained by the method are also bound to lose a sample of the length of the window width. For this reason, Antonakakis et al. (2020) further proposed the time-varying parameter vector autoregressive model (TVP-VAR) [18], which was found to be superior to the VAR-DY model under the sliding time window in terms of measuring the dynamics of volatility correlation in the time series. with the help of the risk contagion analytical framework, which not only portrays the direction, intensity, or correlation of risk contagion in different financial sectors, but also identifies the central sources of risk contagion. Therefore, this risk spillover approach has also gained significant attention. For example, Guo Na (2022) used the TVP-VAR-DY model to study the spillover effects of three markets (Chinese coal, oil and natural gas)with the A-share market [19]. Yang Kun (2024) comparatively analysed the pricing power and dynamic characteristics of China’s crude oil futures and three types of mature crude oil futures by studying the dynamic information spillover effect between global crude oil futures and Asian crude oil spot markets [20]. In conclusion, the TVP-VAR-DY model offers a distinct advantage over other models previously discussed. Unlike these models, the TVP-VAR-DY model is capable of measuring the overall spillover effect in a multidimensional system. Additionally, it can capture the total information spillover degree and information reception degree of each industry in relation to other industries within the system. Furthermore, it can determine the direction and intensity of the information spillover of each industry to other industries. Consequently, the TVP-VAR-DY model will be selected for the purposes of research and analysis in this paper.

Furthermore, more academic research has been conducted to address the role of emergencies in influencing the overall market. Candila V et al. (2021) focuse on the advent of the COVID-19 pandemic in 2020 and examine the interdependence between oil returns and exchange rate movements in oil-exporting and oil-importing countries, concluding that before the COVID-19 pandemic, China, India and South Korea had negative (lagged) correlations, and after the COVID-19 pandemic, the correlations increased for these countries [21]. Later, Zhang Nianhua (2023) investigated the risk spillover effect of international crude oil market volatility on the stock market under unexpected event shocks [22]. Bai Lan (2024) investigated the interrelationship between investor attention and different stock sectors under unexpected events [23]. Shen Yue (2023) employs a U.S. monetary policy perspective to investigate the spillover effects on China’s systemic financial risk during periods of major emergencies [24]. Throughout the existing studies, firstly, the literature on risk spillovers in the financial market mainly focuses on the exchange market, crude oil market and financial market, and the risk measure of the financial sector mostly adopts the first two types of measures, and rarely adopts the third type of measure to study the risk spillover effect within the financial sector. Secondly, the existing literature mostly studies the systematic risk spillover during the epidemic. However, there is rarely any literature on the risk spillover effect between the financial sectors in China before and after the epidemic. This is a significant gap in the literature on financial spillover effects, and it is of paramount importance in preventing and resolving financial risks.

The objective of this paper is to empirically study the risk spillover effect among financial sectors during different periods of the epidemic. To this end, the TVP-VAR-DY model is employed to portray the risk fluctuation among financial sectors (banking, insurance, securities, and multi-finance). The data set was divided into three stages, based on the key points in the epidemic’s development. These stages were defined as follows: the pre-epidemic stage, which encompasses the period before the epidemic’s outbreak, the epidemic stage, which is characterised by the rapid spread of the epidemic and the absence of full liberalisation and normalisation, and the post-epidemic stage, which is defined by the full liberalisation and normalisation of the epidemic. This was done in order to gain a deeper understanding of the specific risk spillover characteristics observed in China’s financial subsectors before and after the epidemic. In addition, in the pre-epidemic stage, this paper further considers the risk spillover effects in the financial market, including the trade war between China and the US, and the P2P platform storms, in order to facilitate comparative analyses with the pre- and post-epidemic stages.

The overall content of the paper is organised as follows: Chapter 0 is the introduction. Chapter 1 is the literature review. Chapter 2 is the model construction and variable selection. Chapter 3 is the empirical analysis. Chapter 4 is the main conclusions and policy recommenda -tions of the paper.

## 3 Overview of theoretical models

### 3.1 TVP-VAR-DY model

In order to better understand the time-varying parameter vector autoregressive spillover model (TVP-VAR-DY), this paper first introduces the fixed-parameter vector autoregressive model, also known as the structural vector autoregressive model (SVAR), as follows.


$$Ay_{t}=F_{1}y_{t-1}+F_{2}y_{t-2}+\ldots \ldots +F_{s}y_{t-s}+\mu_{t},t=s+1,\ldots ..,n$$ (1)

Where, *y*_*t*_ denotes the *k*×1 dimensional vector of observations in each financial subsector i, *A* and *F*_1_,…*F*_*s*_ are the coefficient matrices of *k*×*k*, the random disturbance term *μ*_*t*_ denotes the structural shocks to *k*×1, *μ*_*t*_~*N*(0,∑∑), where ∑ is the diagonal array of *k*×*k*, and *A* is the lower triangular matrix, as follows.


$$A=(\begin{array}{cccc} 1 & 0 & \ldots & 0\\ a_{21} & \ddots & \ddots & \vdots \\ \vdots & \ddots & \ddots & \vdots \\ a_{k1} & \ldots & a_{k,k-1} & 1 \end{array}),\sum =(\begin{array}{cccc} \sigma_{1} & 0 & \ldots & 0\\ 0 & \ddots & \ddots & \vdots \\ \vdots & \ddots & \ddots & \vdots \\ 0 & \ldots & 0 & \sigma_{k} \end{array})$$

Where *σ*_*i*_(*i* = 1,…,*k*) denotes the standard deviation of the structural shock.

After matrix transformation, Eq (1) can be converted into the form of VAR model, as follows.


$$y_{t}=B_{1}y_{t-1}+B_{2}y_{t-2}+\ldots \ldots +B_{t-s}y_{t-s}+A^{-1}\sum \varepsilon_{t},t=s+1,\ldots ..,n$$ (2)

Where, *B*_*i*_ = *A*^−1^*F*_*i*_, for *i* = 1,….,*s*, *ε*_*t*_~*N*(0,*I*_*k*_). Furthermore, the row elements in the matrix B are replaced by *k*^2^*s*×1 dimensional vectors. Define $X_{t}=I_{k}⊗(y_{t-1}^{'},\ldots .,y_{t-k}^{'})$, where Eq (2) can be simplified to.


$$y_{t}=X_{t}\beta +A^{-1}\sum \varepsilon_{t}$$ (3)

It is obvious that all the variable parameters in Eq (3) are fixed in time and cannot portray the time-varying characteristics of the parameters among the variables. Therefore, the time-varying model that can capture the variable parameters is further constructed, i.e., the TVP-VAR-DY model that is to be used for the empirical analyses in this paper. Based on Eq (3), the TVP-VAR-DY model is denoted as.


$$y_{t}=X_{t}\beta_{t}+{A_{t}}^{-1}\sum_{t}\varepsilon_{t},\;t=s+1,\ldots .,n$$ (4)

Where *β*_*t*_, *A*_*t*_, ∑_*t*_ are time-varying parameters. Furthermore, the elements of matrix *A*_*t*_ are rearranged into a column vector by eliminating zeroes and ones, as follows.

$$a_{t}=(a_{21},a_{31},\ldots ,a_{k1})'$$ (5)

and let

$$h_{t}=(a_{1t},a_{2t},\ldots ,a_{kt})'$$ (6)

then

$$h_{jt}=\log\sigma_{jt}^{2},j=1,2,\ldots ..,k;t=s+1,\ldots .,n$$ (7)

The time-varying parameters in the model are assumed to satisfy the stochastic wandering process.


$$\begin{array}{c} \beta_{t+1}=\beta_{t}+\varepsilon_{\beta t}\\ a_{t+1}=a_{t}+\varepsilon_{at}\\ h_{t+1}=h_{t}+\varepsilon_{ht} \end{array},\;(\begin{array}{c} \varepsilon_{t}\\ \varepsilon_{\beta t}\\ \varepsilon_{at}\\ \varepsilon_{ht} \end{array})∼N(0,(\begin{array}{cccc} I & O & O & O\\ O & \sum_{\beta } & O & O\\ O & O & \sum_{a} & O\\ O & O & O & \sum_{h} \end{array})),\;t=s+1,\ldots .,n$$ (8)

Where, $\beta_{s+1}∼N(\mu_{\beta_{0}},\sum_{\beta_{0}}),a_{s+1}∼N(\mu_{a_{0}},\sum_{a_{0}}),h_{s+1}∼N(\mu_{h_{0}},\sum_{h_{0}})$, and three time- varying parameters *β*_*t*_, *a*_*t*_, *h*_*t*_ are uncorrelated. Also, assume that ∑_*β*_, ∑_*a*_, ∑_*h*_ are all diagonal matrices. Let $y={\left\{y_{t}\right\}}_{t=1}^{n}$, *ω* = (∑_*β*_, ∑_*a*_, ∑_*h*_). According to the generalised VAR model framework proposed by Diebold and Yilmaz, the idea of variance decomposition is applied to obtain the generalised forecast error variance decomposition (GFEVD) for the H-step, which can be expressed as follows:

(ΘH)j,k=σkk−1∑h=0H−1((Ψh∑)j,k)2∑h=0H−1(Ψh∑Ψh')j,j (9)

Where Ψ_*h*_ denotes the N×N dimensional moving average coefficient matrix of lag order h, with *σ*_*kk*_ = (∑)_*kk*_, (Θ_*H*_)_*j*,*k*_ denotes pairwise directional spillovers from k to j. The impact of sector k on sector j is accounted for through forecast error variance shares. When *j*≠*k*, (Θ_*H*_)_*j*,*k*_, also known as the cross variance share, or spillover effect, denotes the forecast error variance of forecast sector j due to shocks in sector k. Furthermore, these variance shares are normalised such that each row sums to 1, which implies that all sectors explain 100 percent of the forecast error variance of sector j, i.e.


$${\left(\Theta_{H}^{'}\right)}_{j,k}=\frac{{\left(\Theta_{H}\right)}_{j,k}}{\sum_{k}{\left(\Theta_{H}\right)}_{j,k}}$$ (10)

Where, (Θ’_*H*_)_*j*,*k*_ denotes the pairwise directional spillover between sector k and sector j after normalisation. The total correlation is defined as the share of variance contributed by cross- prediction errors other than own prediction errors. It is calculated as the ratio of the sum of the non-diagonal terms (∑_*j*≠*k*_(Θ’_*H*_)_*j*,*k*_) to the sum of the integer terms (∑(Θ’_*H*_)_*j*,*k*_) in the normalised variance decomposition matrix, with the denominator being the cumulative effect of all shocks and the numerator being the cumulative effect of one shock in sector j, i.e.


$$C_{H}=100\frac{\sum_{j\neq k}{\left(\Theta {'}_{H}\right)}_{j,k}}{\sum {\left(\Theta {'}_{H}\right)}_{j,k}}=100(1-\frac{Tr\{\Theta {'}_{H}\}}{N})$$ (11)

Where *Tr*{.} is the trace function. Thus, the total spillovers refer to the relative contribution of other elements of the VAR system to the forecast variance. k can be used to measure the degree of total spillovers across the financial sector (shorthanded as TOTAL), which indicates how shocks in one sector affect other sectors. Firstly, the directional spillovers received by sector j from sector k, referred to as the degree of total directional spillovers received from other sectors (abbreviated as FROM), i.e.


$$C_{H,j\leftarrow •}=100\frac{\sum_{k,k\neq j}{\left(\Theta {'}_{H}\right)}_{j,k}}{\sum {\left(\Theta {'}_{H}\right)}_{j,k}}=100\frac{\sum_{k,k\neq j}{\left(\Theta {'}_{H}\right)}_{j,k}}{N}$$ (12)

Secondly, the sum of directional spillovers passed on from sector j to all other sectors k (abbreviated as TO) must be measured, i.e.


$$C_{H,•\leftarrow j}=100\frac{\sum_{k,k\neq j}{\left(\Theta {'}_{H}\right)}_{k,j}}{\sum {\left(\Theta {'}_{H}\right)}_{k,j}}=100\frac{\sum_{k,k\neq j}{\left(\Theta {'}_{H}\right)}_{k,j}}{N}$$ (13)

Finally, the calculation subtracts the impact of other sectors on industry j from the impact of industry j on all other sectors, i.e., TO minus FROM, to obtain the net spillover measure (abbreviated as NET), as follows:

$$C_{H,j}=C_{H,•\leftarrow j}-C_{H,j\leftarrow •}$$ (14)

Where, *C*_*H*,*j*_ denotes the difference between the overall spillover value that sector j contributes to other sectors and the spillover value it receives from other sectors. If *C*_*H*,*j*_ is positive, it means that sector j has a greater impact on the other sectors in the financial system than the other sectors have on themselves and is a spillover of risk. Conversely, if *C*_*H*,*j*_ is negative, it means that sector j has a lesser impact on the other sectors in the financial system than the other sectors have on themselves and is a receiver of risk.

### 3.2 Selection of variables

This paper takes the financial sector as its background, and is based on the "Provisions on the Standard Regulations on the Delineation of Enterprises in the Financial Sector", which were jointly researched and formulated by the People’s Bank of China, the China Banking Regulatory Commission (CBRC), the China Securities Regulatory Commission (CSRC), the China Insurance Regulatory Commission (CIRC), and the National Bureau of Statistics (NBS). The study selects the four major sectors of the financial sector as the object of study, including the banking sector, insurance sector, securities sector, and multi-finance, which specifically cover four major categories, including monetary and financial services, capital market services, insurance sector, and other financial sectors. With regard to the four major sectors in the sample, this article respectively selects the daily returns of the CSI Bank Index, CSI All-Share Industry Index, CSI Founder Fubon Insurance Theme Index, and Multi-Financial Index to measure. The sample interval is from 20 June 2017 to 31 December 2023, encompassing significant events, such as the U.S.-China trade war, the withdrawal of P2P platforms from the market due to economic turbulence, the U.S.’s money market events, and the emergence of the novel coronavirus, so as to better analyse the dynamic variability of outbreaks on risk spillovers. The data were obtained from the Wind database. According to the development process of the epidemic, the sample interval can be roughly divided into three stages, as shown in Table 1 (All datasets are detailed in S1–S3 Datasets).

**Table 1:** Sample stage division.

stage	time interval	basis of division
**pre-epidemic**	2017.6.20–2019.12.31	On the one hand, considering the occurrence of financial events such as the US-China trade war and the centralised withdrawal of P2P online lending platforms in 2018, people’s panic spread and caused financial market turbulence. Meanwhile, the outbreak of the New Crown Epidemic at the beginning of 2020, Wuhan announced the sealing off the city on 23 January in order to prevent the further spread of the epidemic. Considering that before sealing the city, it was in the pre-spring festival period, the flow of people was large, and people’s panic was serious. In light of the aforementioned considerations, this paper proposes an extension of the data set to 2017. This will enable further exploration of the spillover effects of the financial mega-event on various financial sectors, with a view to facilitating comparison with the New Crown epidemic.
**in-epidemic**	2020.1.02–2022.12.8	Given the relatively brief period of time that Wuhan was sealed off, it is not treated as a standalone stage. Instead, this stage can be roughly divided into two main periods. ①The period of rapid spread following the outbreak of the epidemic: the period of Wuhan’s sealing of the city. This was a crucial juncture in the epidemic’s trajectory, as it coincided with a rapid spread of the virus within Wuhan. People all over the country realised the seriousness of the matter and worked together to fight against the epidemic. Finally, on April 8, 2020, after a gap of 76 days, Wuhan was unsealed. ②The period of non-comprehensive liberalisation of normalisation following the unsealing of Wuhan: After the unsealing of Wuhan, the epidemic continued to spread throughout the country, and entered a more than two-year-long stage of normalisation of epidemic prevention and control through occasional nucleic acid testing, trip codes, place codes, quarantine and other forms.
**post-epidemic**	2022.12.9–2023.12.31	After 8 December 2022, the entire country (with the exception of Hong Kong, Macao and Taiwan) will no longer carry out full nucleic acid screening, and the implementation of the willingness to test as much as possible. This marks the advent of a new era of full liberalisation of the norm, which can be considered a significant victory for the national protest movement.

Above all, this paper focuses on the dynamic risk spillover changes in various sectors and the risk contagion effect among sectors at different stages. This is done in order to analyse more clearly the stage-by-stage changes in the risks of different financial sectors and the characteristics of the differences in the course of China’s successful anti-epidemic stage. In response to some emergencies, it provides investors and regulators with certain reference bases and makes some policy recommendations.

### 3.3 Descriptive statistical analyses

In order to reduce the heteroskedasticity between the data and to make the data smoother, logarithmic differencing was applied to the data in this paper. The logarithmic yield r_t_ was obtained to observe the change in the yield of the data, i.e.


$$r_{t}=\ln(p_{t})-\ln(p_{t-1})$$ (15)

Where P_t_ denotes the stock price at moment t. The specific results are shown in Table 2.

**Table 2:** Descriptive statistical analysis of the total sample.

	Sector name	Mean	Std.	Min	Max	Skewness	Kurtosis	JB	Qstatistic	ADF
**Stage 1**	Banking	0.00	0.01	-0.04	0.06	0.33	5.58	183.30***	1.06	-8.28***
	Securities	0.00	0.02	-0.10	0.10	0.34	8.69	849.14***	1.05	-7.92***
	Insurance	0.00	0.02	-0.07	0.09	0.19	4.98	105.66***	1.05	-8.29***
	Multifinance	0.00	0.02	-0.09	0.09	0.29	8.18	704.33***	1.07	-7.65***
**Stage 2**	Banking	0.00	0.01	-0.07	0.09	0.32	7.22	541.32***	1.02	-9.23***
	Securities	0.00	0.02	-0.11	0.09	0.30	6.67	409.83***	1.05	-9.16***
	Insurance	0.00	0.02	-0.09	0.09	0.35	5.87	259.37***	1.03	-9.11***
	Multifinance	0.00	0.02	-0.11	0.07	-0.46	8.92	854.05***	1.04	-9.25***
**Stage 3**	Banking	0.00	0.01	-0.03	0.04	0.42	4.94	48.29***	1.05	-6.60***
	Securities	0.00	0.01	-0.04	0.07	0.86	6.73	180.98***	1.09	-6.00***
	Insurance	0.00	0.01	-0.03	0.06	0.92	5.22	89.17***	1.08	-6.39***
	Multifinance	0.00	0.01	-0.04	0.04	0.24	3.56	5.81***	1.11	-6.52***

As can be seen from Table 1, the difference in the mean values among the financial subsectors in different periods is not large, and they all fluctuate around the zero value. By analysing the standard deviation, the maximum value and the minimum value, it can be clearly seen that the fluctuation of the financial market is larger during the pre-epidemic and mid-epidemic periods, and the fluctuation of the financial market is relatively smoother in the post-epidemic period, which suggests that the epidemic had a certain degree of impact on the stability of the financial market. Furthermore, when analysing the different sub-sectors within the same period, the banking sector is relatively the most stable, while the other sub-sectors do not vary much in terms of volatility. Comparing the different periods, in general, the banking sector remains the smoothest across the three periods. From the perspective of skewness and kurtosis, each financial sector exhibits sharp peaks and thick tails in different periods, and basically shows a right-skewed distribution. The JB statistic test shows that the logarithmic return series of all subsectors do not conform to a normal distribution. The Q statistic also shows that none of them has autocorrelation under the condition of lagging by 12 orders. Lastly, the ADF unit root test demonstrates that all the logarithmic return series are stationary. Consequently, the next step of the analysis can be carried out.

## 4 Empirical results and analyses

This paper presents a systematic study of risk contagion and spillover patterns among financial sectors in the pre-, mid- and post-event stages of the epidemic. The outbreak of the new coronavirus provides the research background for this study. The paper is developed from three aspects. Firstly, the gross spillover index between subsectors is analysed from a static perspective to examine the overall spillover effect at different stages of the epidemic. Secondly, the intensity and direction of gross spillovers, the intensity and direction of net spillovers from subsectors, and the pairwise net spillovers between two and two sectors of the financial system are analysed from a dynamic point of view, so as to examine the spillover effects of each subsector on other financial subsectors at different stages of the epidemic. Finally, different lag orders are selected to test the robustness of the empirical results of the model.

### 4.1 Analysis of static spillovers

As can be seen from Tables 3–5, the total system spillover index in the first stage reaches 57.86%, and the overall spillover intensity of the financial sector is 231.44. The total system spillover index in the second stage reaches 61.28%, which indicates that the outbreak of the epidemic has created a significant disruption in the financial sector. In the third stage, due to the victorious end of the fight against the epidemic, the socio-economics gradually starts to recover, and the total system spillover index is 56.93%, showing a decreasing trend, but since economic recovery needs a long period of adjustment, therefore, as of March 2024, the total spillover index has decreased but not significantly. Overall, the total spillover of the system demonstrates an initial increase followed by a decline within the three stages of the epidemic development.

**Table 3:** Static spillovers in the first stage (pre-epidemic).

	Banking	Securities	Insurance	Multi-finance	FROM
**Banking**	43.67	15.83	28.35	12.15	56.33
**Securities**	15.02	40.26	16.86	27.87	59.74
**Insurance**	27.53	17.13	41.82	13.51	58.18
**Multi-finance**	12.77	29.87	14.54	42.81	57.19
**To**	55.33	62.83	59.75	53.53	231.44
**NPSO**	98.99	103.09	101.58	96.34	**TCI 57.86**
**NET**	-1.01	3.09	1.58	-3.66	

Note: ‘FROM’ denotes the total risk contagion received by an industry from other industries; ‘TO’ denotes the total risk spillover from an industry to other industries; ‘NET’ denotes the intensity of the net spillover from an industry to other industries; and ‘TCI’ in the lower-right corner of the table denotes the total spillover effect of the system at that stage, as in Tables 4 and 5.

**Table 4:** Static spillover effects in the second stage (in the epidemic).

	Banking	Securities	Insurance	Multi-finance	FROM
**Banking**	40.95	16.39	28.89	13.77	59.05
**Securities**	15.10	37.57	20.50	26.83	62.43
**Insurance**	25.88	19.90	36.66	17.55	63.34
**Multi-finance**	13.24	28.2	18.87	39.69	60.31
**To**	54.22	64.5	68.26	58.16	245.14
**NPSO**	95.17	102.07	104.92	97.84	**TCI 61.28**
**NET**	-4.83	2.07	4.92	-2.16	

**Table 5:** Static spillover effects in stage 3 (post epidemic).

	Banking	Securities	Insurance	Multi-finance	FROM
**Banking**	43.34	16.22	27.47	13.07	56.76
**Securities**	15.00	41.94	17.37	25.68	58.06
**Insurance**	26.34	18.01	41.89	13.76	58.11
**Multi-finance**	12.62	27.50	14.66	45.22	54.78
**To**	53.96	61.73	59.50	52.52	227.71
**NPSO**	97.20	103.67	101.39	97.74	**TCI 56.93**
**NET**	-2.80	3.67	1.39	-2.26	

From the perspective of the FROM and TO, in the first stage, due to the outbreak of significant financial events, the financial market is volatile. It can be found that the securities sector is most likely to receive the impact of risk spillovers from other sectors. Simultaneously, the securities sector is also prone to infecting other sectors with its own risks. This is consistent with the empirical evidence. In the second stage, the outbreak of the epidemic resulted in a significant increase in insurance claims, placing considerable strain on the insurance sector. With a subsequent increase in risk, and thus the insurance sector became the main risk spillover at that stage. In the third stage, the epidemic came to an end and the economy began to recover. At this juncture, the securities sector was the largest risk spillover, and with the secondary outbreak of the epidemic, the insurance sector continued to represent the largest risk receiver. In addition, when analysed from the NET perspective, the net spillover indices of the banking and multi-financial sectors are negative in all three stages, indicating that these two sectors are relatively stable and receive more risk contagion from other sectors. The insurance sector, like the total systemic spillover index, exhibits an increasing and then decreasing trend, with its and the securities sectors being the main spillover of risk.

The following section will examine the dynamic overflow of these three phases in greater detail.

### 4.2 Analysis of dynamic spillover effects

#### 4.2.1. Analysis of total system spillovers

**Fig 1:**
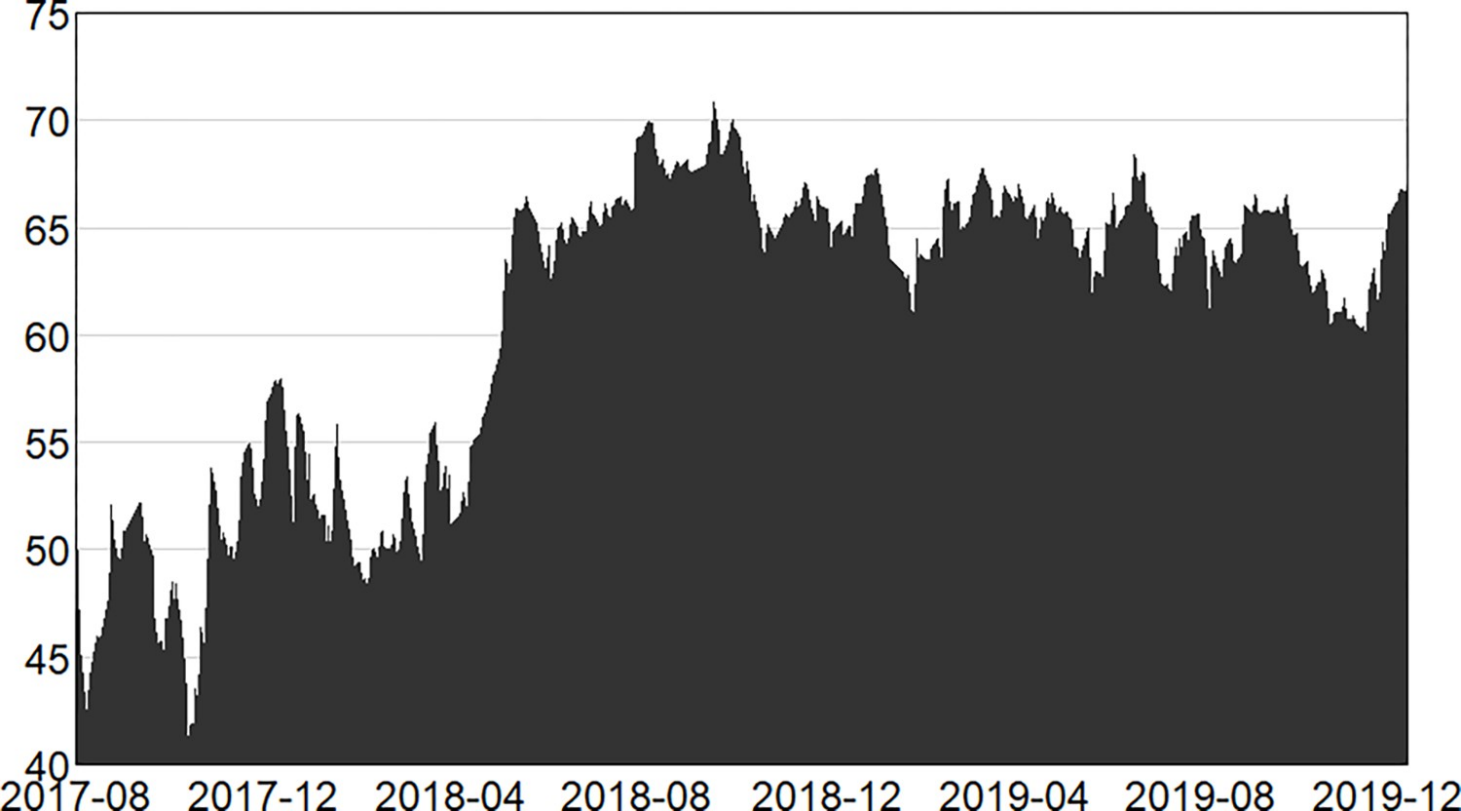
Changes in total spillage during the first stage (pre-epidemic).

**Fig 2:**
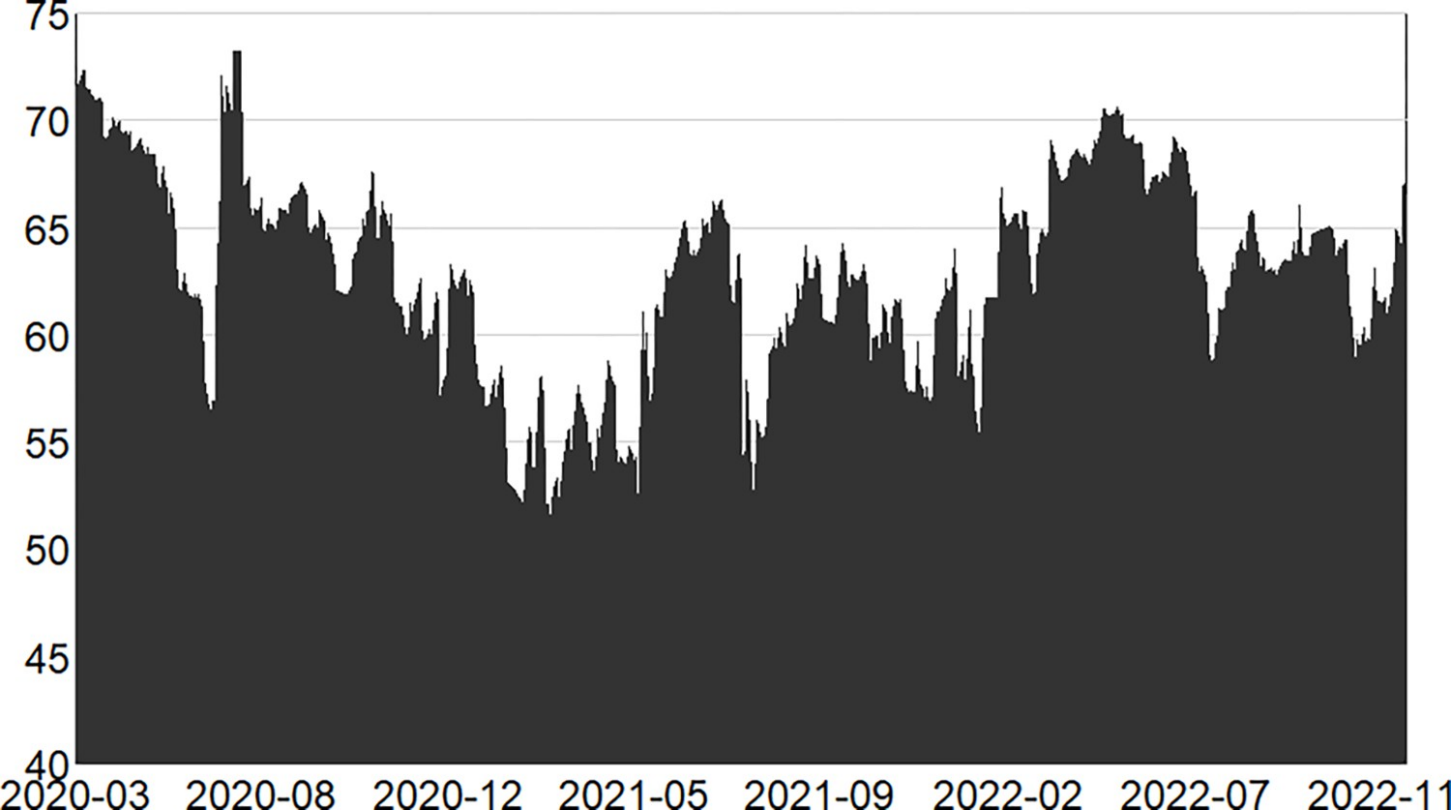
Changes in total spillage during the second stage (in-epidemic).

**Fig 3:**
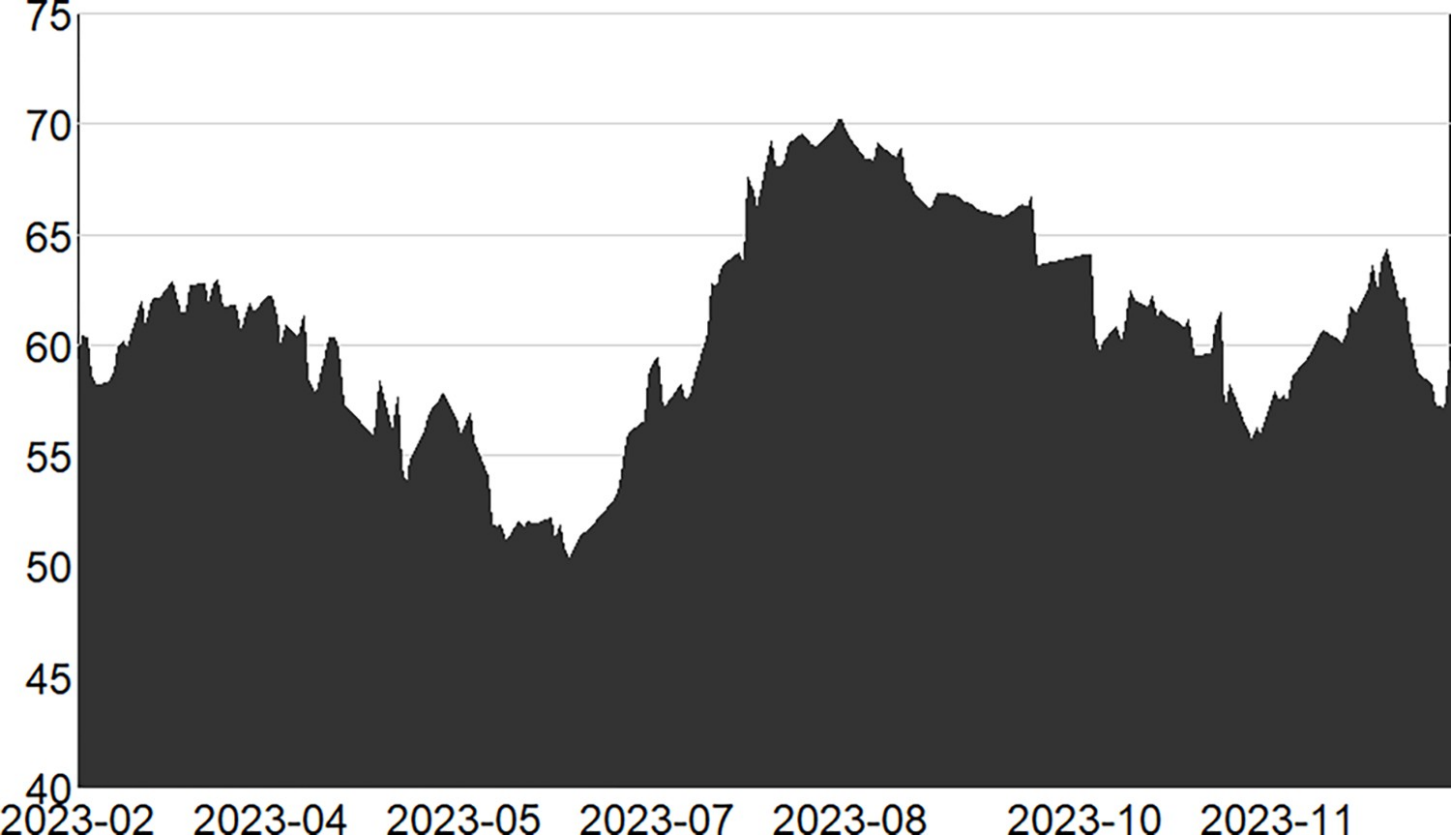
Changes in total spillage during the third stage (post epidemic).

Figs 1–3 illustrate the dynamics of the total system overflow in the pre-, mid- and post-epidemic stages.

In the first stage, before the outbreak of the epidemic, the overall volatility of the financial market was in line with the external environment. The market as a whole was relatively stable before 2018, but the latter half of 2018 witnessed a shift, with a series of financial occurrences exerting a considerable influence on the market, resulting in a discernible surge in overall market volatility and a prolonged period of heightened spillover intensity. For example, the trade war between China and the United States in 2018, which lasted for 18 months, resulted in a decline in the export trade volume of the two countries while imposing constraints on their economic development. Additionally, the advent of peer-to-peer (P2P) platforms that started in 2018 stormed one after another until all of them retreated from the event, which triggered the continuous turbulence in China’s financial market. This was followed by the money market event in September 2019, which was characterised by a considerable number of U.S. treasury bond issuances, leading to a decline in the level of reserves and significant volatility in interest rates in the repo market.

In the second stage, the full-scale outbreak of the epidemic in early 2020, Fig 2 clearly demonstrates that the fluctuation is more intense than before and that the intensity of spillover has increased. In January 2020, the epidemic was revealed, causing a rise in public panic. This was followed by the closure of Wuhan, which led to a notable increase in the total spillover risk. With the lifting of the closure of Wuhan, there was a brief period of weakening, but the epidemic soon spread to all parts of the country, the total spillover risk intensity suddenly increases. But soon the epidemic spread throughout the country, resulting in a sudden surge in the total spillover risk intensity. At this time, there was social disorder and continued turmoil in the financial markets. During this stage, the majority of factories and enterprises throughout the country shut down and suspended production, leading to disruptions in the industrial chain and shortages in the supply chain. This led to a notable decline in business production and consumption activities, accompan -ied by a surge in unemployment. The offline business of banks was also hit hard, the securities and stock markets were in turmoil, and the performance of numerous companies was adversely affected by the epidemic, with their share prices declining markedly. In particular, sectors such as tourism, airlines and hotels were most severely affected, with the share prices of many listed companies falling below record lows. Concurrently, the highly contagious nature of the new coronary pneumonia has led to a large number of people being infected and even hospitalised for quarantine and treatment. During this period, insurance companies have incurred a substantial burden in terms of claims. As successive waves of the epidemic emerged, the sense of crisis and uncertainty intensified, directly impacting the stability of the financial market. Subsequently, a series of epidemic prevention and restriction policies were introduced by the state, and the new crown epidemic was gradually controlled. From 2021 onwards, the overall epidemic across the country was sporadic and disseminated. At the same time, in order to prevent large-scale gathering of people, online scenario business appeared and was facilitated to accelerate the process of digitisation and automation. This contributed to the gradual restoration of economic and social structures, accompanied by a notable decline in the risk of spillover. However, there were still localised gatherings due to the emergence of disseminated cases epidemics, including several more large-scale epidemic infections around 2022, so the total spillover risk fluctuates more sharply in this stage.

The third stage, the epidemic entered the normalized management stage in the first half of 2023, but there are still some aggregated outbreaks, such as the second wave of the epidemic that began in May 2023, which lasted about two months and basically reached universal immunity. To this point, the new crown of the epidemic is close to the end of the epidemic, marking the people’s resistance to the epidemic of the overall victory. At this time, the community has resumed work and production in a significant number of areas. However, in comparison to the period of economic downturn precipitated by the previous epidemic, the general economic environment has demonstrated improvement. Nevertheless, the economy continues to confront a multitude of uncertainties and challenges, it will take a long time to fully recover. The overall total spillover risk exhibited a reduction in intensity, but the intensity of the weakening is not large.

#### 4.2.2. Analysis of the direction and intensity of sector spillovers

Where, ryh denotes the banking sector, rzq denotes the securities sector, rbx denotes the insurance sector, and rdy denotes the multi-financial sector.

**Fig 4:**
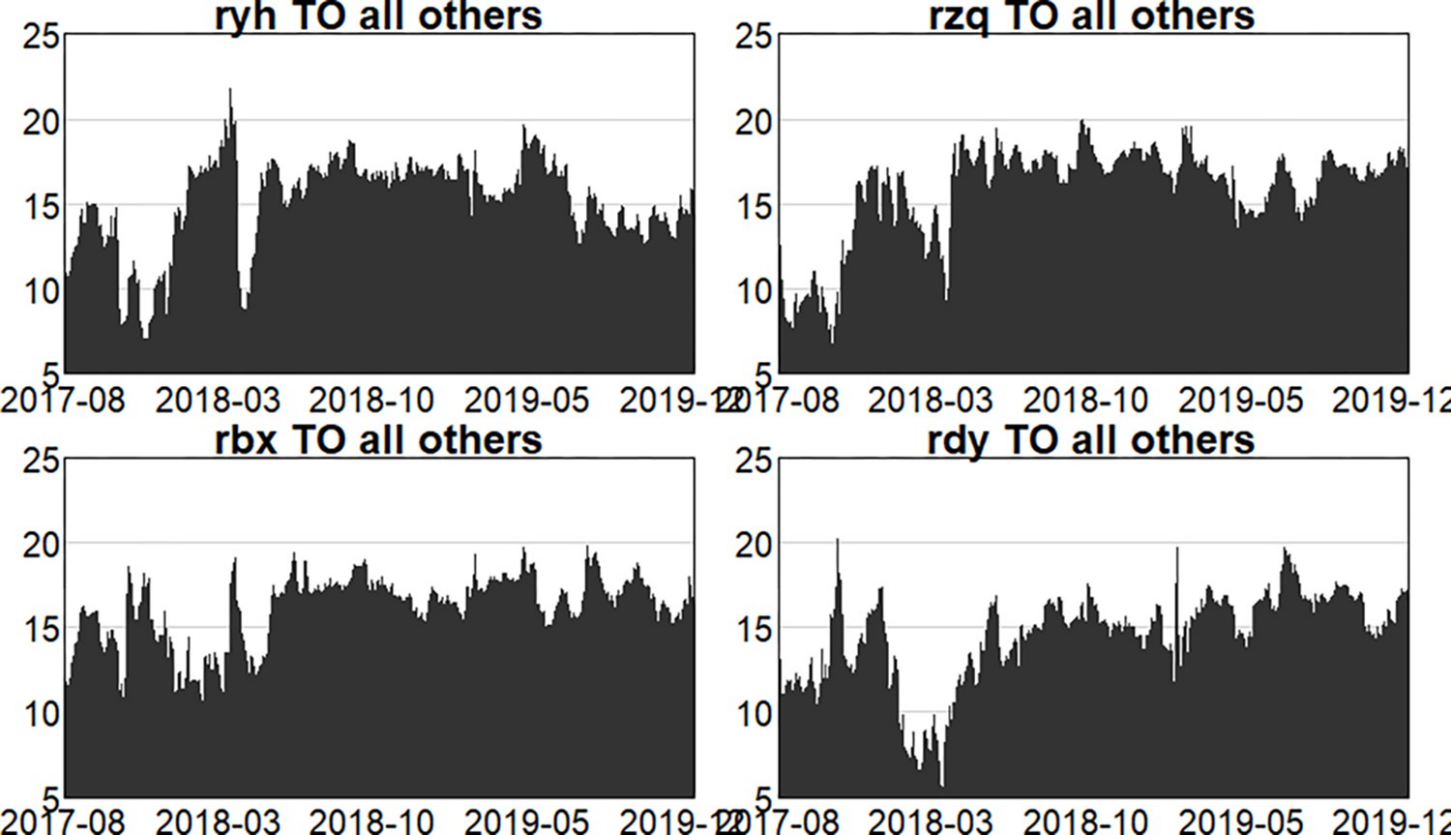
Analysis of TO dynamics (pre-epidemic).

**Fig 5:**
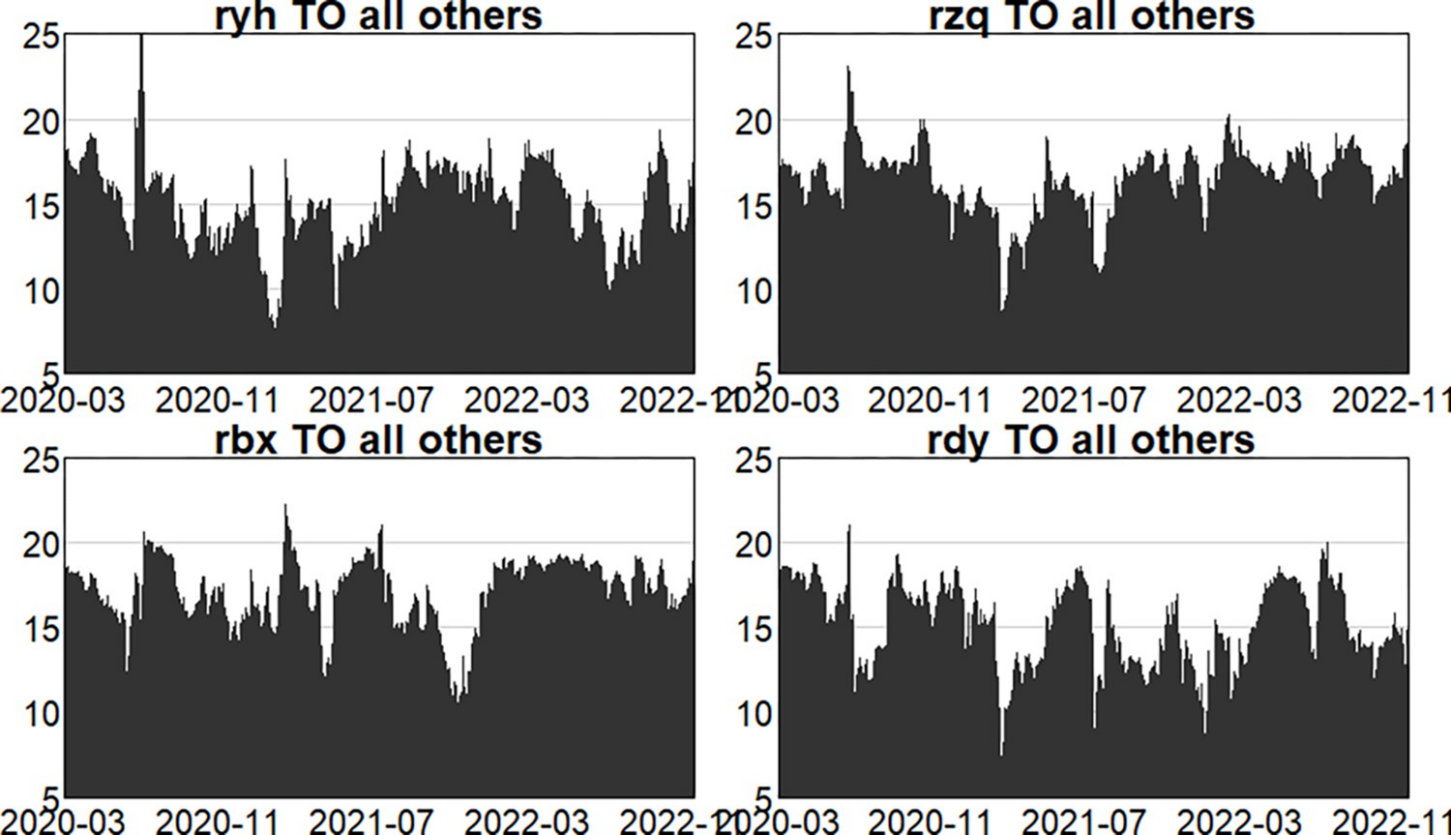
Analysis of TO dynamics (in-epidemic).

**Fig 6:**
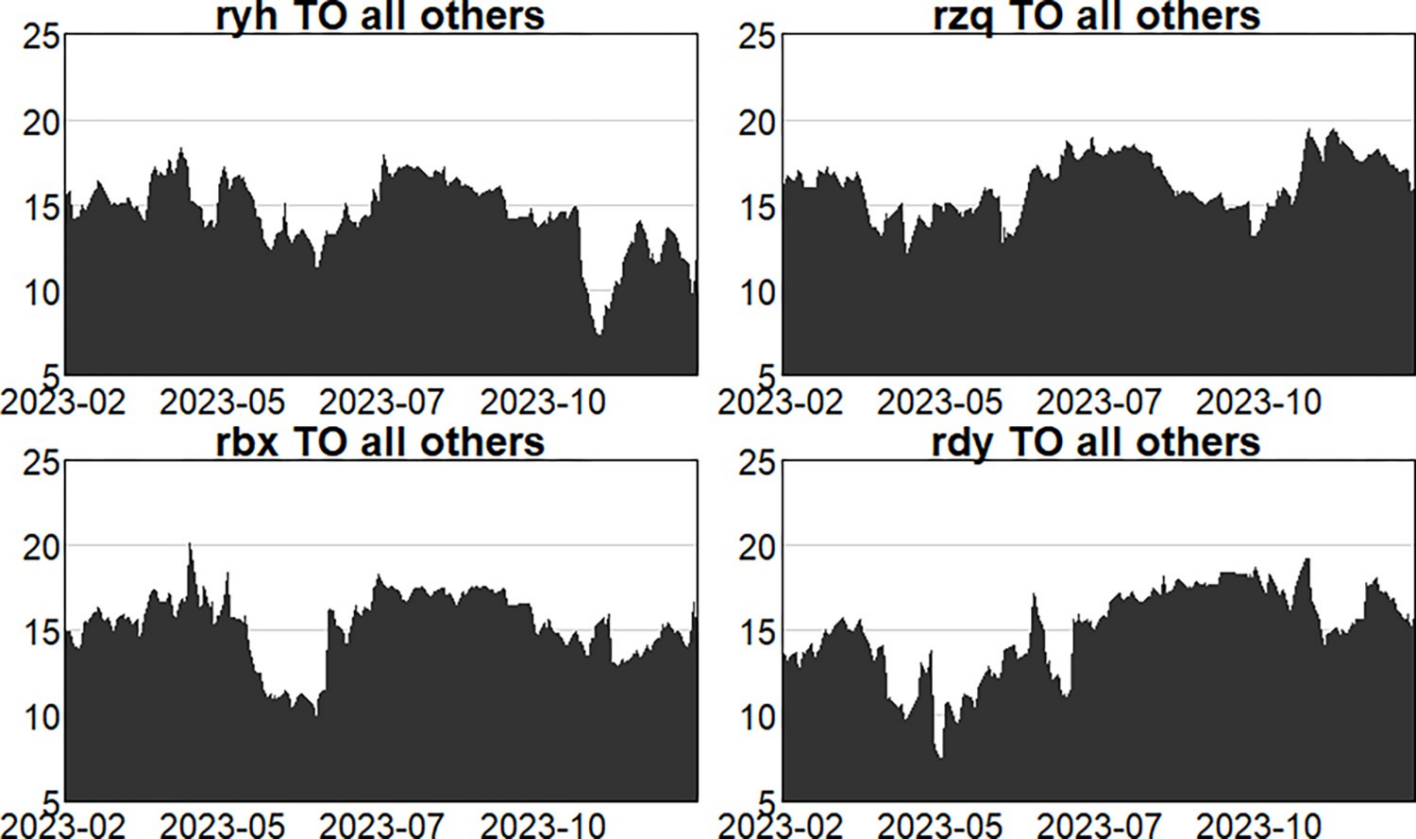
Analysis of TO dynamics (post epidemic).

**Fig 7:**
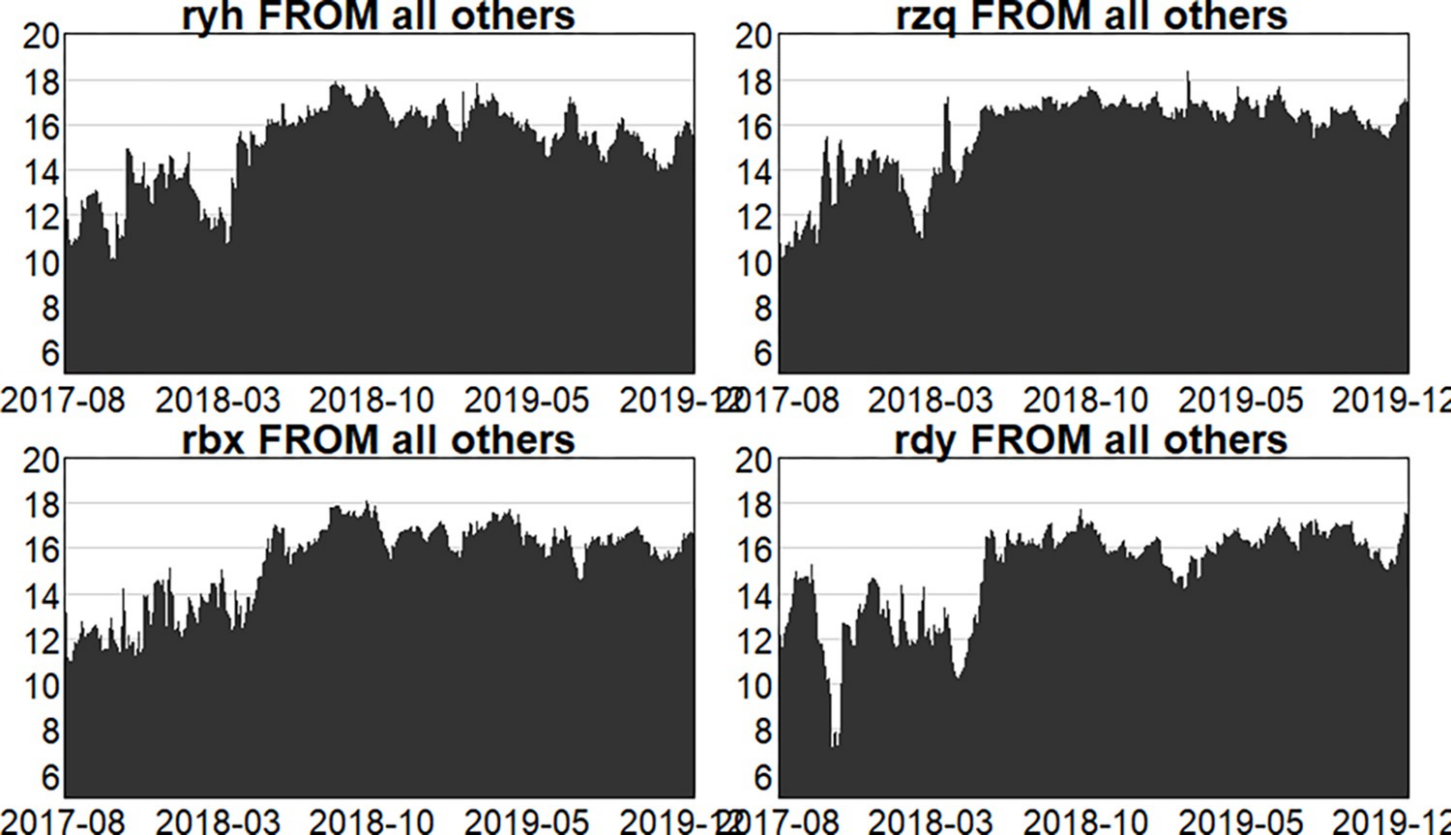
Analysis of FROM dynamics (pre-epidemic).

**Fig 8:**
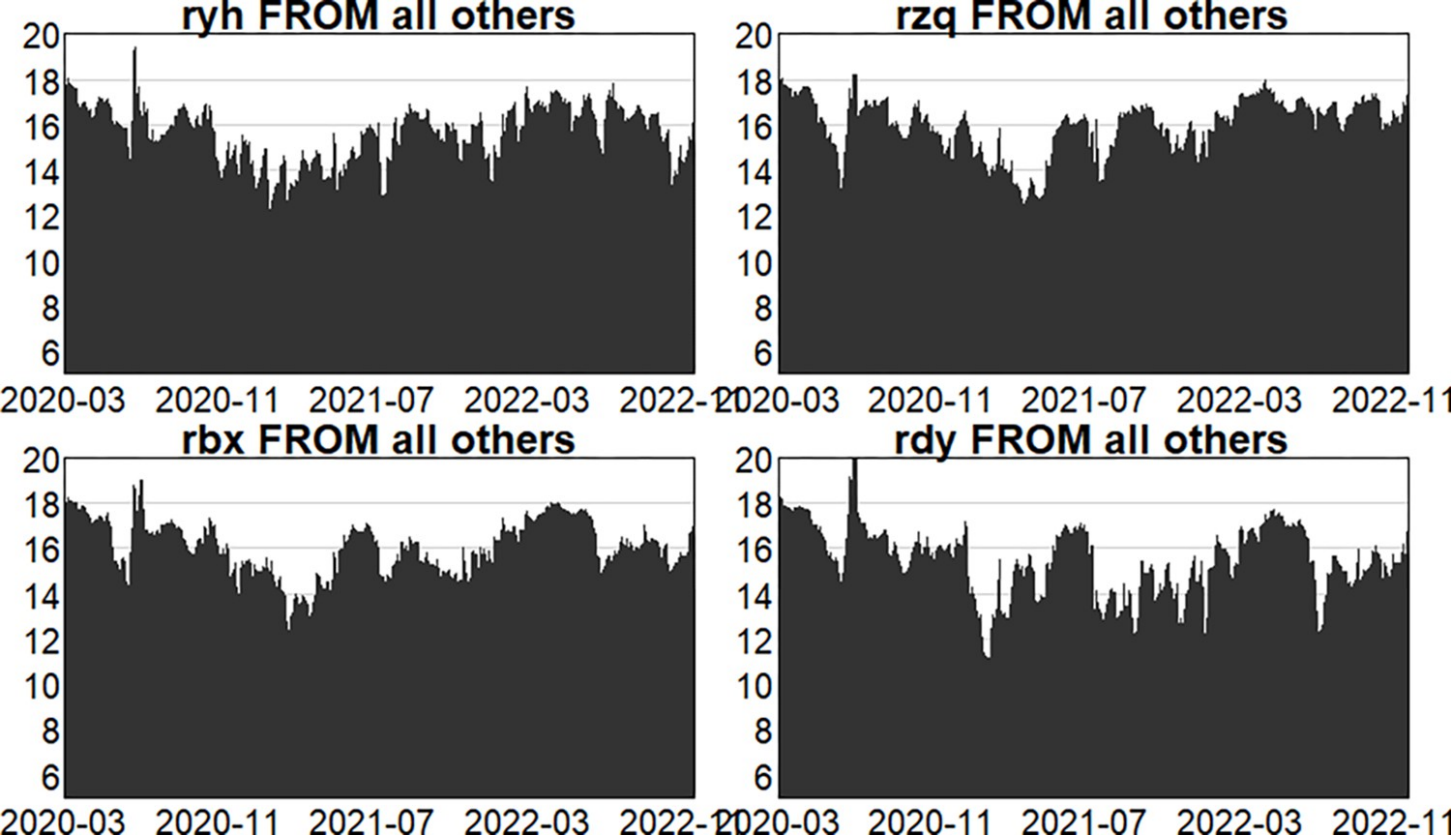
Analysis of FROM dynamics (in-epidemic).

**Fig 9:**
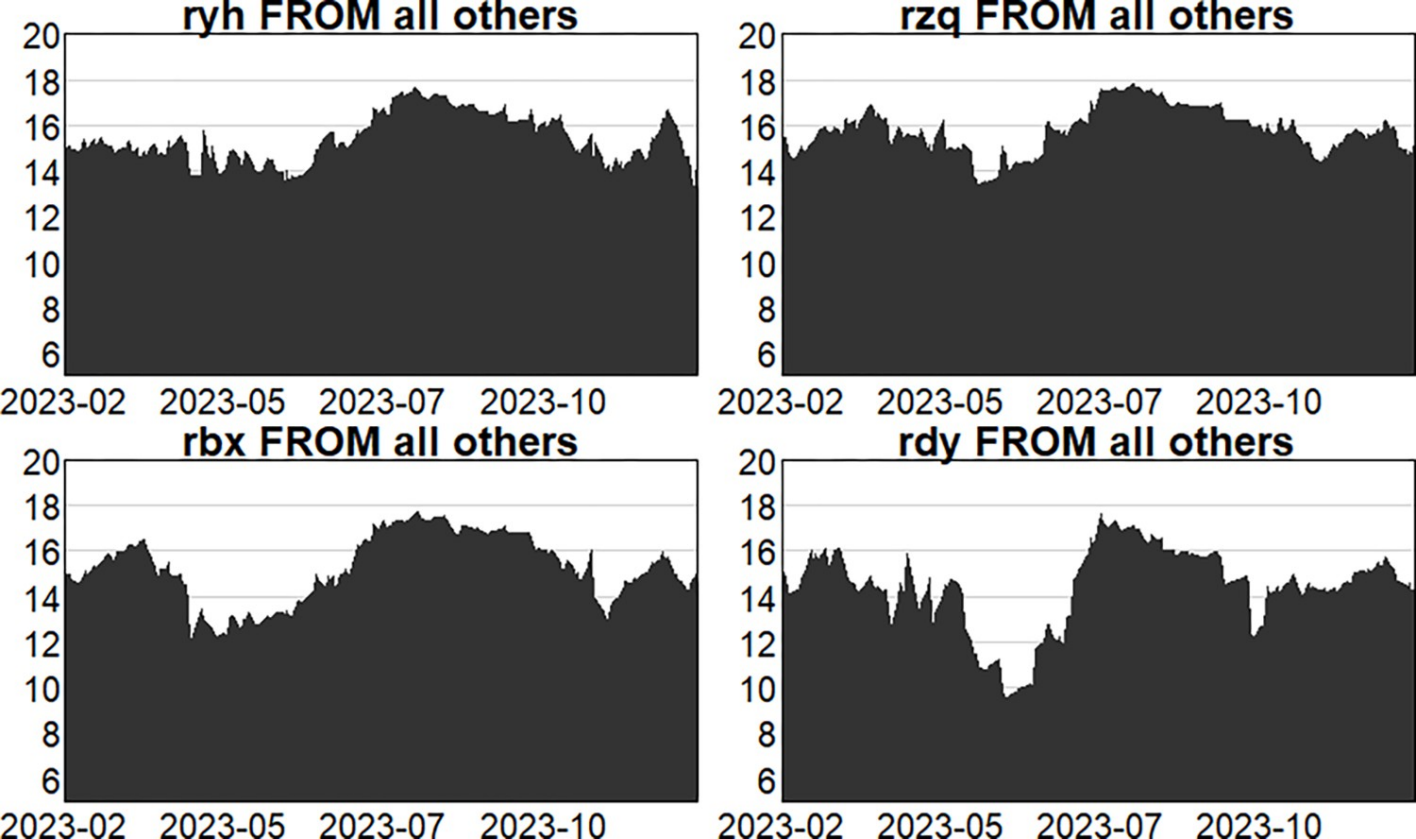
Analysis of FROM dynamics (post epidemic).

Figs 4–6 show the dynamic risk spillover (TO) from one sector to the others. Unsurprisingly, in the first stage, commencing in the second half of 2018, the intensity of total spillover risk from one sector to the others increased significantly due to the frequency of financial events. In the second stage, the volatility oscillations of the four major sectors are more intense due to the recurring nature and uncertainty of the epidemic, and the risk spillover intensity of the securities and insurance sectors to the rest of the market is higher. Respectively, and the volatility is more intense in the first and second stages. In the third stage, the volatility is relatively smoother. Figs 7–9 show the dynamic risk spillover (FROM) received by a sector from other sectors. It can be observed that the securities sector and the insurance sector are also susceptible to risk spillovers from other sectors, while the banking and multi-financial sectors are relatively less affected, but with more violent fluctuations.

#### 4.2.3. Analysis of the dynamics of net spillovers

**Fig 10:**
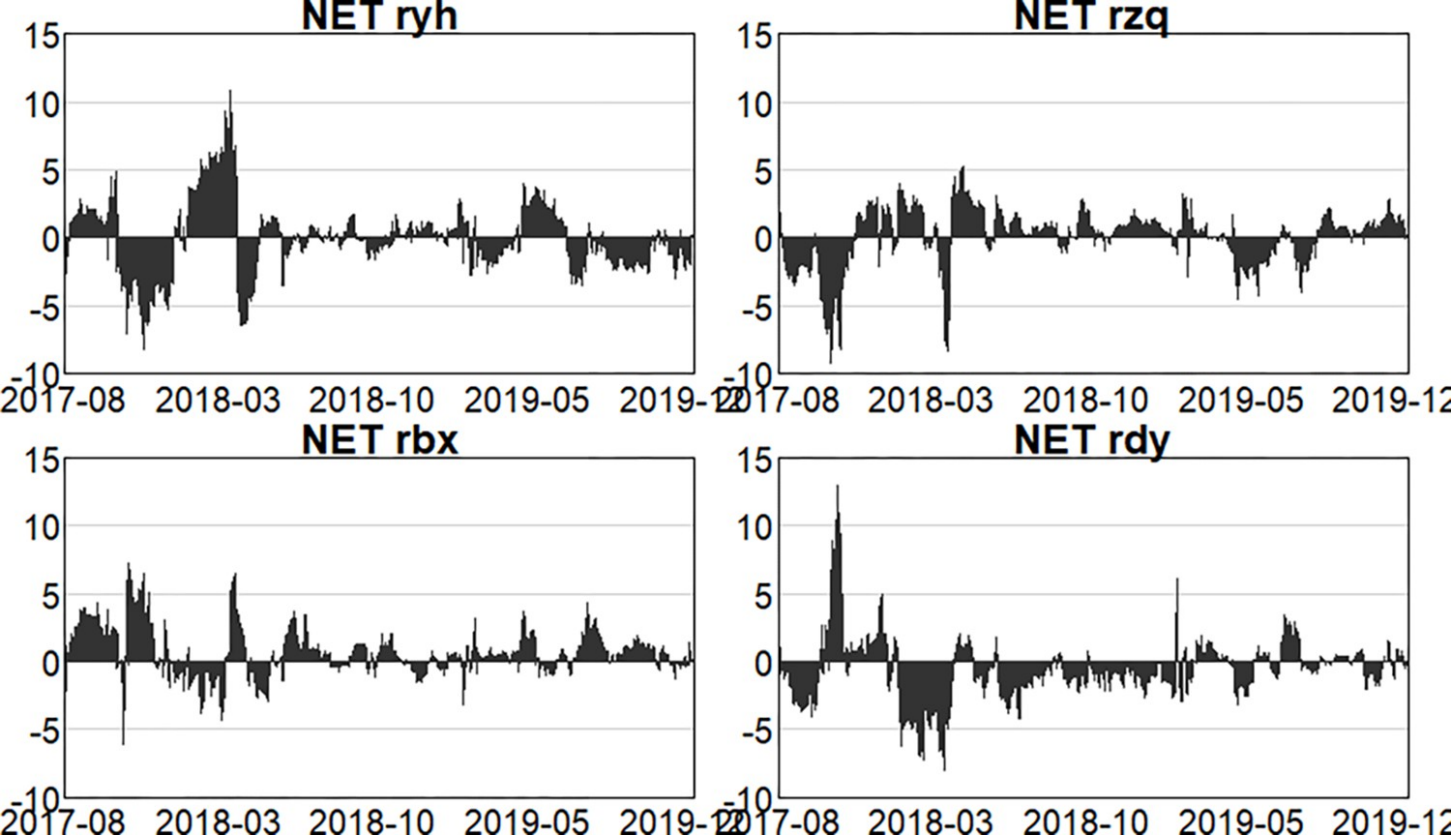
Dynamics of net spillage in the first stage (pre-epidemic).

**Fig 11:**
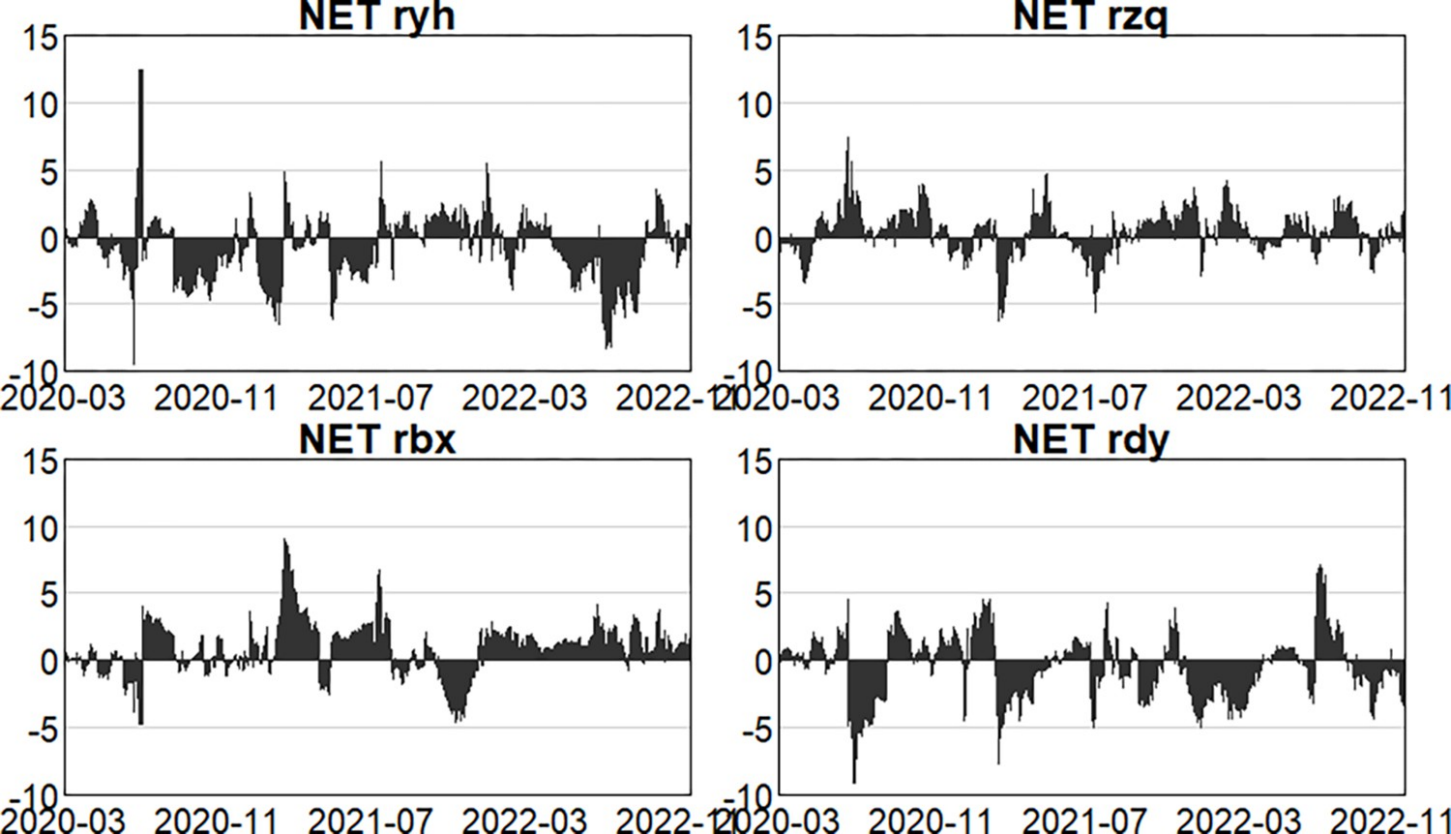
Dynamics of net spillage in the second stage (in-epidemic).

**Fig 12:**
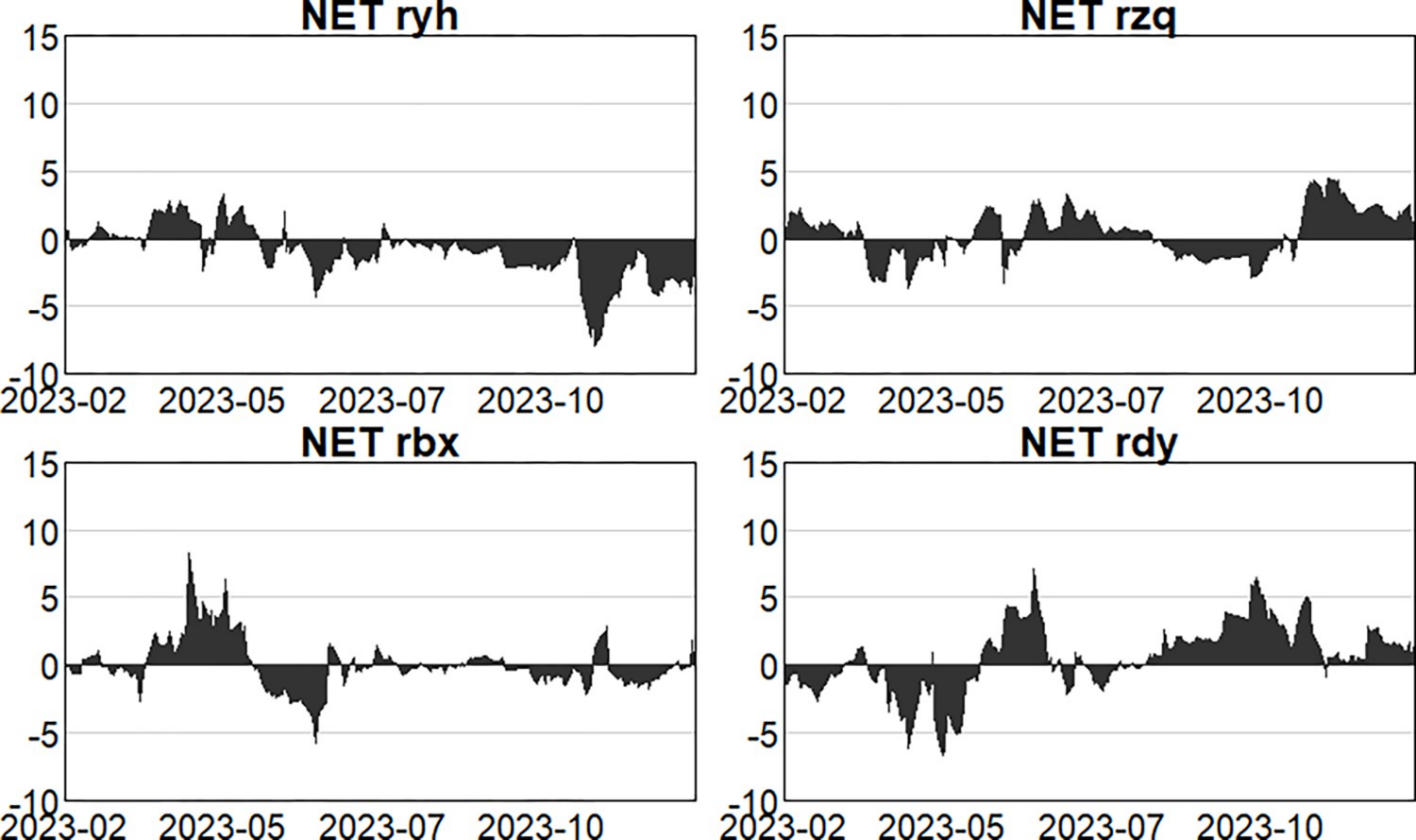
Dynamics of net spillage in the third stage (post epidemic).

Furthermore, Figs 10–12 illustrate the changing dynamics of net spillovers. Firstly, in the pre-epidemic stage, before March 2018, risk spillovers and receipts from the four main sectors kept changing, and in general, the banking and insurance sectors were net risk spillovers, and the securities and multi-financial sectors were net risk receipts. Then, due to the events such as the US-China trade war and the 2019 money market, the securities and insurance sectors became net risk spillovers, the multi-financial sector and the banking sector gradually shifted to become net risk receivers. Secondly, during the outbreak stage, the banking and multi-financial sectors are clearly more net risk receivers, and the banking sector is the main net risk receiver. For the insurance sector, the proportion of insurance payouts also changs in stages due to the constant repetition of the outbreak stages, resulting in the overall dynamics of the insurance sector show a repeated alternation of net risk receivers and spillovers, which is a net risk spiller in general, and the securities sector is also a net risk spiller in this stage. Finally, in the post-epidemic normali -zation stage, by May 2023, NPL ratios within banks and insurance claims are rising as a result of the epidemic, resulting in the banking and insurance sectors being net spillovers of risk and the securities, and multi-finance sectors being net receivers of risk, but unsurprisingly the intensity of spillovers is less than in the epidemic. In the second outbreak in May 2023, when immunization was universal and of short duration, the securities and multi-finance sectors were the main net risk spillovers, and the banking and insurance sectors were the main net risk receivers, with the intensity of the receipts from the banking sector being greater. Since October 2023, due to the sharp rise and fall in US Treasuries, long considered one of the world’s safest assets, and resulting in sharp fluctuations in the global financial market, the net risk spillover intensity of the securities sector and the multifinancial sector has increased. As a main net risk receiver, the intensity of the banking sector has also increased, with a relatively small impact on the insurance sector.

#### 4.2.4. Pairwise net spillover analysis between sectors

**Fig 13:**
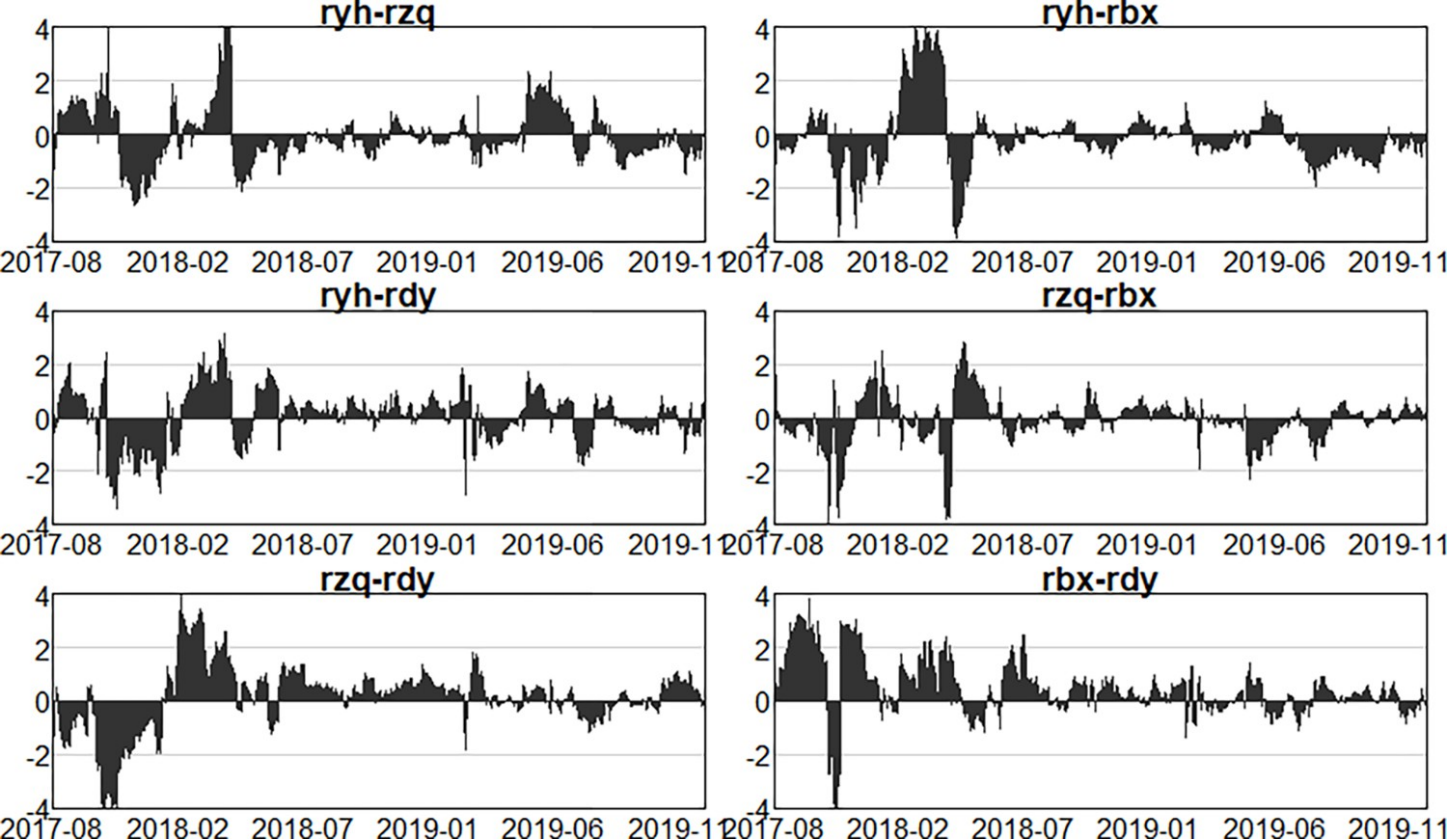
Dynamics of pairwise net spillovers between sectors in the first stage (pre-epidemic).

**Fig 14:**
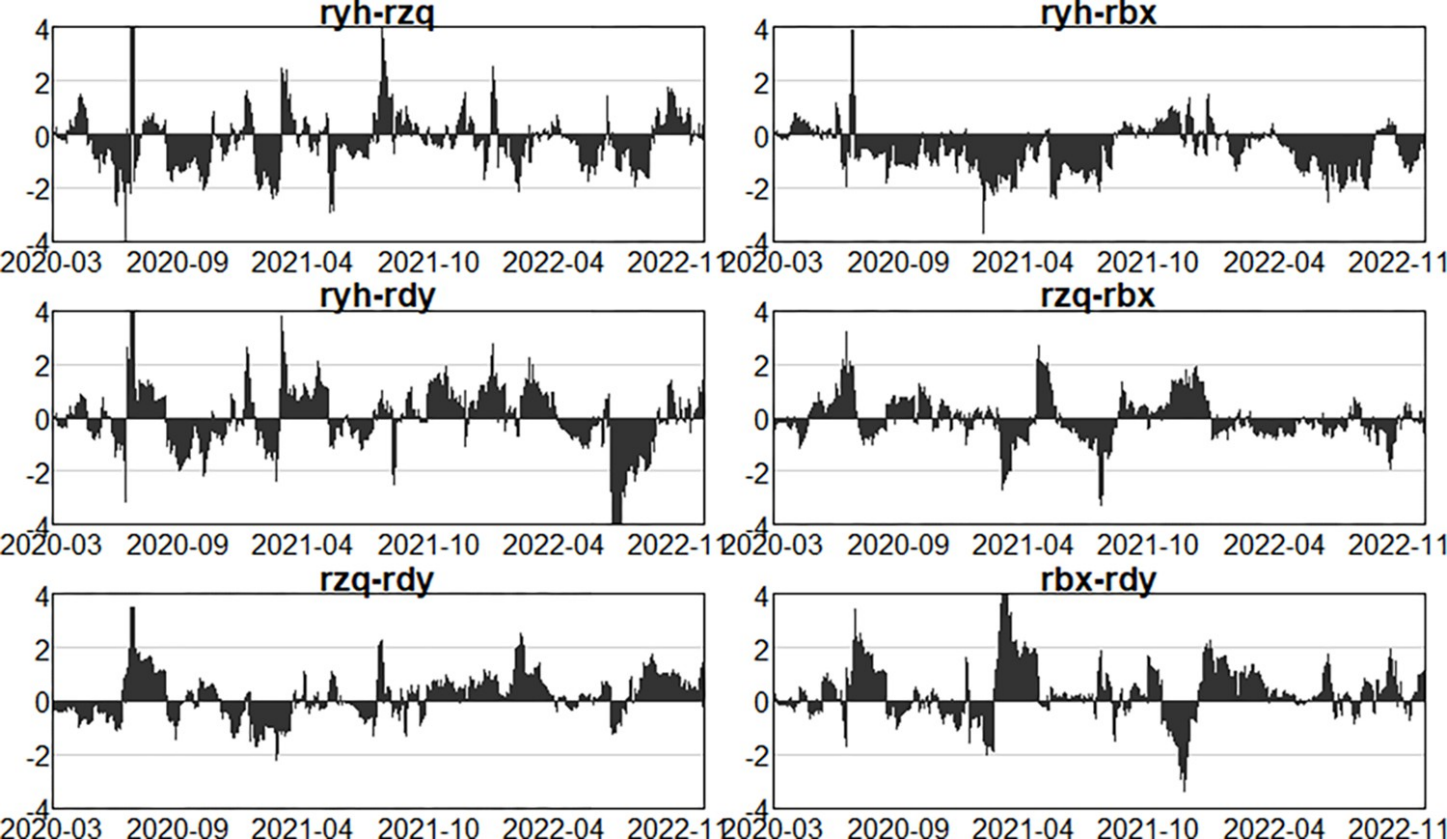
Dynamics of pairwise net spillovers between sectors in the second stage (in- epidemic).

**Fig 15:**
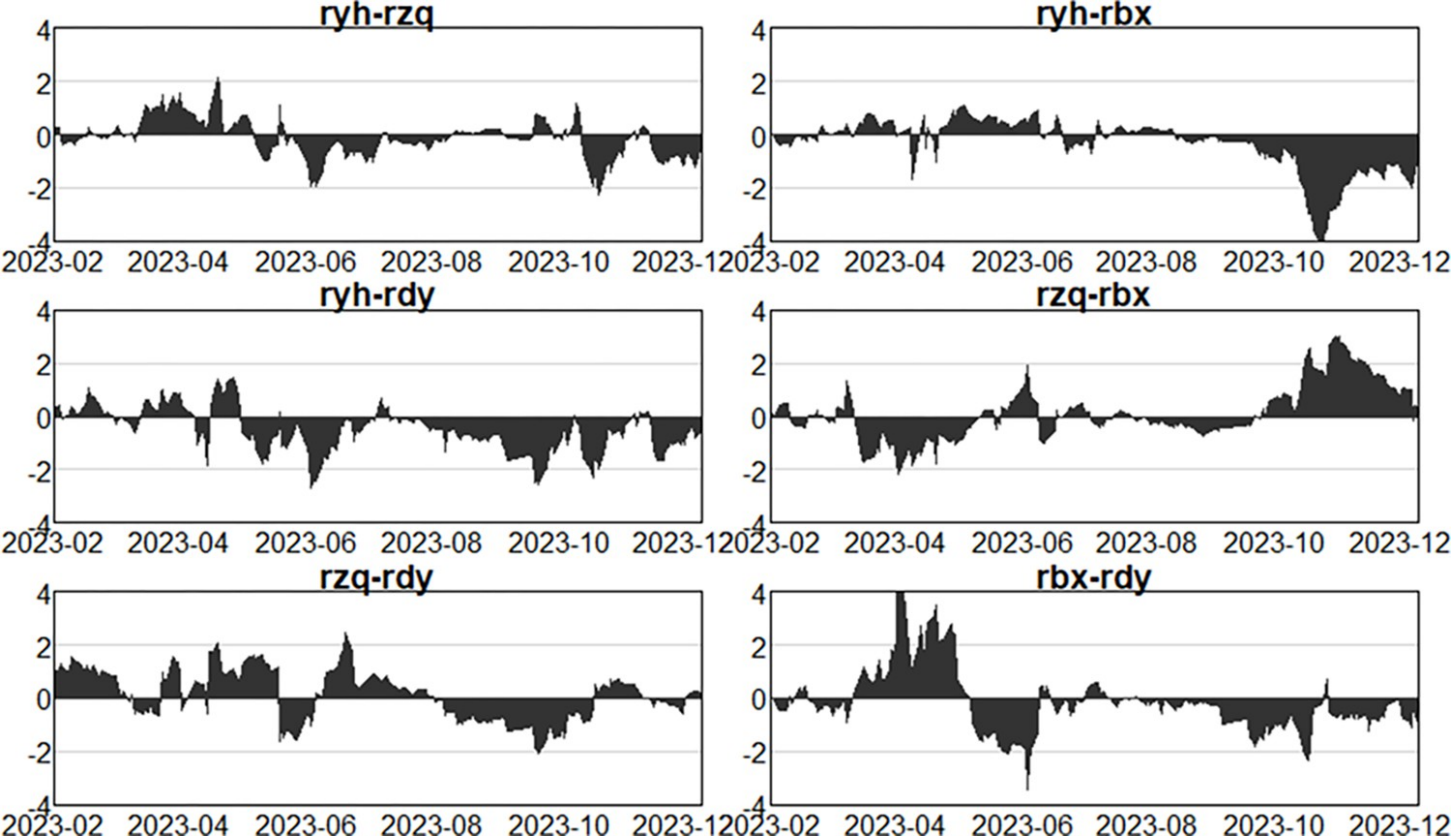
Dynamics of pairwise net spillovers between sectors in the third stage (post epidemic).

Having analysed the net spillovers from one sector to other sectors as a whole above, this section further examines the net spillovers in pairs between two sectors, so as to understand the intensity and direction of risk spillovers between the two sectors, as well as the issue of the variability of risk spillovers at different stages of the process. As shown in Figs 13–15.

Figs 13–15 illustrate the net spillover effects of pairs between the two sectors. First, the banking sector has generated risk spillovers to all three other sectors. Before the epidemic, the banking sector started with positive spillovers to the securities sector, then gradually shifted to negative spillovers, and the overall phenomenon has alternated between positive and negative. In general, banks have been mainly net receivers of risk for both the securities and insurance sectors, while for the multifinance sector, the banking sector has been more of a net spiller of risk. In the outbreak of the epidemic, the banking sector to the securities sector and the insurance sector is mainly shown as a net receiver of risk, that is, more received the risk contagion from the other three sectors, and the securities sector has a greater impact on the banking sector. While the banking sector and the securities sector are more positive and negative alternately, at this point in time, due to the large-scale outbreak of the New Crown Epidemic, the ongoing social unrest, and the epidemic showed aggregated and intermittent outbreaks, resulting in banks receiving more intense risk intensity and volatility in the other three sectors. In the aftermath of the epidemic, although the New Crown epidemic is close to the end, but the economic situation is still not optimistic, all walks of life continue to recover, at this time, the securities, tinsurance and the multi-finance sectors are still a big risk. Therefore, in this period, the banking sector still mainly behaves as a net receiver of risk to the other three sectors, but the risk volatility is significantly much slower and the risk intensity is reduced than in the outbreak, but in November 2023, the intensity of risk spillover from the insurance sector and the diversified financial sector to the banking sector is significantly stronger due to the US Treasury bond spike and crash event, and in general. In all the three periods, due to the leverage ratio of the banks, business model, etc., which gives banks more cushion to mitigate risk in the event of an outbreak, have not changed much in terms of their own risk, and have mainly acted as net recipients of risk in the financial system, despite the impact of the New Crown Epidemic.

Second, comparing the two sectors that is securities and insurance, before the epidemic, the net spillover intensity between the two was larger before March 2018, but according to the previous dynamic spillover analysis, the total spillover intensity in that period was actually not very large, considering that it was due to the impact of the sector’s own characteristics, the overall volatility of the financial market in line with the external environment, and the dynamics between the two in the second half of the period, the securities sector being mainly as a net recipient of risk, the intensity of risk spillover between the two was roughly between -1 and 1, with no sharp fluctuations. In the epidemic outbreak stage, it can be clearly seen that the two still show positive and negative alternation, and fluctuations are more intense, the risk spillover intensity becomes larger, at this time the whole securities market is very unstable, turbulence. In the epidemic, the securities sector mainly as a net receiver of risk, the intensity of the spillover becomes significantly weaker, but after October 2023, the securities sector has become a net spillover of risk.

Next, analysing the net risk spillovers between the securities sector and the multifinance sector, it is easy to see that the securities sector has acted more as a risk spillover to the multifinance sector in all three periods. This indicates that the securities sector has been more prone to transmit risk to the multifinance sector.

Finally, to analyze the net risk spillover between the insurance sector and multi-finance, before the epidemic, the insurance sector is basically a net risk spillover. Following the outbreak of the epidemic, with the continuous change of the epidemic, the insurance sector and multi-finance also show the phenomenon of risk contagion and risk spillover alternating with each other, and the intensity becomes bigger, the fluctuation is also more intense. However, the insurance sector continued to act as a net risk spillover. After the epidemic, the economy began to recover, the proportion of claims dropped sharply, and the riskiness of the insurance sector was reduced, but the financial market was still highly uncertain, and the risk of multi-finance itself was still very high, so multi-finance was mainly a net risk spillover.

### 4.3 Robustness tests

**Fig 16:**
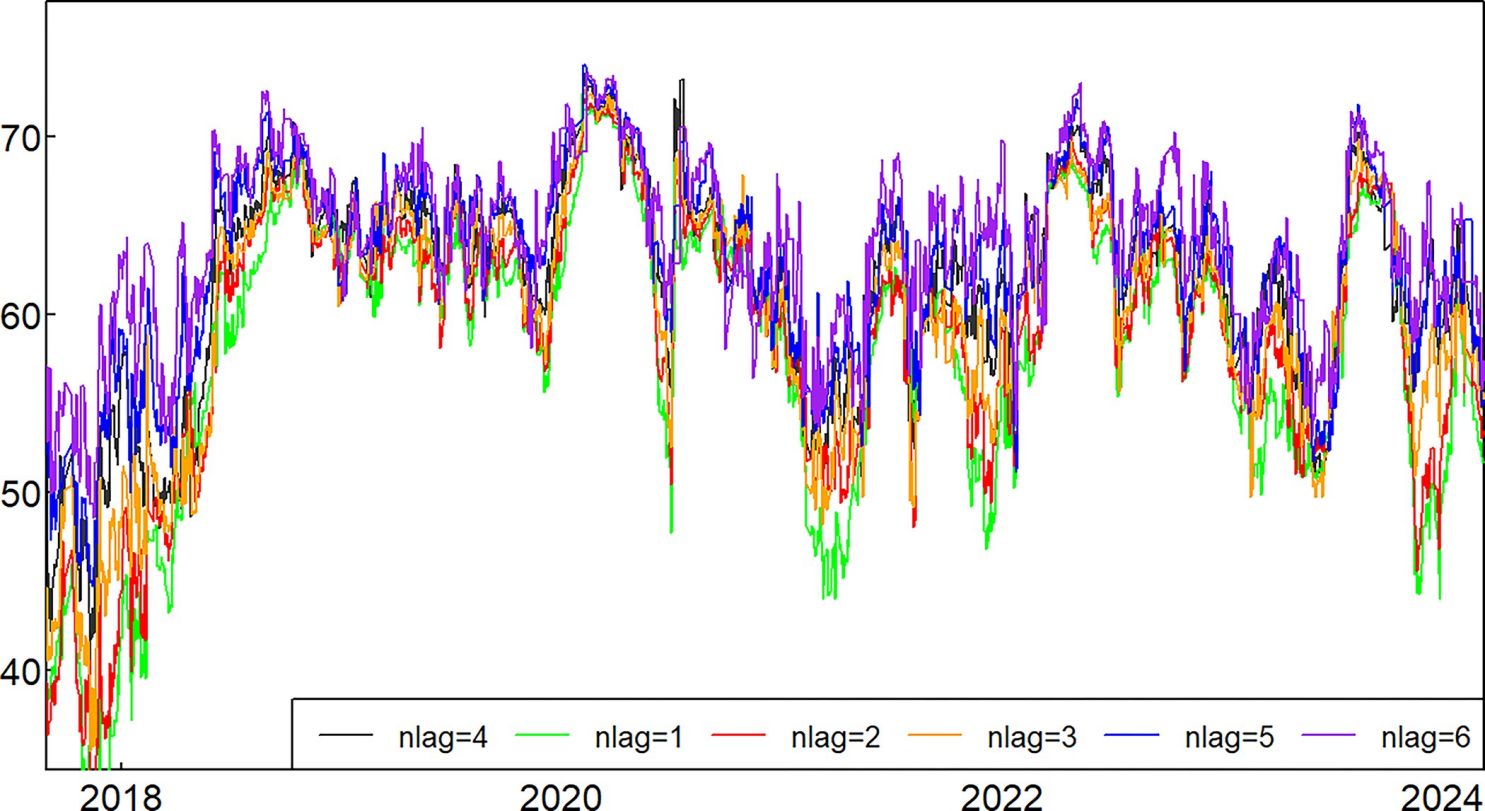
Comparison of total overflow for different lag orders.

In order to verify the robustness of the empirical results, this paper adopts the method of replacing the lag order of the TVP-VAR model for a robustness test and extends the sample time to 31 January 2024 (S4 Dataset). In the empirical part of the previous paper, the lag order used in this paper for the TVP-VAR model was 4th order. In the robustness test, this paper further constructed TVP-VAR models of 1st, 2nd, 3rd, 5th and 6th order, calculated the dynamic total spillover indexes under different orders respectively, and made a comparison with the preceding paper. The specific index changes are shown in Fig 16.

Fig 16 illustrates that the total indices exhibit minimal variation over time, with a consistent trend. This validates the model’s efficacy and demonstrates its independence from the number of lag orders and the selection interval of the sample.

## 5 Conclusions and recommendations

### 5.1 Conclusions

Using the TVP-VAR-DY model, this paper investigates the dynamic risk spillover characteristics of the four major financial sectors (banking, securities, insurance, and multi- financial) in different stages of the New Crown epidemic during the period between 20 June 2017 and 29 December 2023. The data is divided into three stages: pre-, mid-, and post-epidemic. The principal findings of the study are as follows.

Firstly, the risk spillover of financial subsectors is affected by the New Crown epidemic, which is manifested by the variability of risk spillover in different periods. Secondly, analyzing from the perspective of static spillover, the total spillover of the system shows a trend of increasing and then decreasing. On the whole, the banking sector and the multi-finance sector are the main risk receivers, the securities sector has been a risk spillover, the insurance sector has been the risk in the first two periods and a considerable amount of time after the epidemic spillover and finally became a risk receiver. Thirdly, analyzing from the perspective of dynamic total spillover, the financial subsector was clearly affected by the new crown epidemic and was more sensitive to emergencies. After the outbreak of the epidemic, the volatility of the entire market exhibited a notable intensification, with a marked increase in the overall intensity. Fourthly, analyzing from the direction and intensity of industry net spillovers, when the outbreak occurs, the securities and insurance sectors are more sensitive and susceptible to spillovers to other sectors. These, in turn, are more susceptible to the impact of other sectors, and the intensity is much stronger in the epidemic than before and after the epidemic. Fifthly, analyzing from the perspective of pairwise net spillovers between sectors, first of all, the banking sector has been a net receiver of risk to all other sectors and has not experienced much change in its own risk. Then, the securities sector, which was more affected by the New Crown epidemic, has been a net spillover of risk for the banking and multifinancial sectors. To the insurance sector, which was a net recipient of risk at the beginning, and then became a net spillover of risk after the outbreak of the epidemic, with a tendency of increasing and then decreasing in intensity. Finally, between the insurance sector and the multifinancial sector, the insurance sector was a net spillover of risk before and during the outbreak, and then gradually changed to a net receiver of risk. In addition, this paper can also be found, the eruption of financial crises and emergencies should not be underestimated, as they have the potential to significantly impede the stable evolution of the financial market, thereby engendering sustained social unrest.

### 5.2 Recommendations

In light of the aforementioned conclusions, this paper proposes the following recommendations.

Firstly, for each financial subsector, there are significant differences in their business models, characteristics of capital flows and asset structures. Therefore, it is necessary to take into account the characteristics of one’s own sector and formulate some contingency plans in a timely manner, so as to prevent large fluctuations in the sector when emergencies break out. For example, the banking sector, as the core of the financial system, is funded mainly by various types of deposits, and the use of funds is focused on the granting of loans. In the face of emergencies, there may be a rapid rise in the risk of defaults on corporate and individual loans. Therefore, the banking sector should formulate capital deployment and risk disposal programmes for different default rate scenarios, and may take measures such as appropriately extending repayment periods and adjusting loan interest rates. The securities sector is more obviously affected by emergencies and is mainly a risk spiller. Emergencies can lead to substantial and drastic fluctuations in the entire securities market, at which time investor confidence is undermined, so the securities sector prefers to formulate contingency plans from the perspective of its clients to stabilise market expectations and safeguard the order of trading, preventing the emergence of market risks and even the contagion of other sectors. The risk characteristics of the insurance sector are different from the first two in that it operates primarily on risk, and through the collection of premiums, bears the risk of losses that may be incurred by the insured. Following the occurrence of unexpected events, the pressure of insurance claims will suddenly increase. Insurance companies need to establish a sound risk assessment model in normal times in order to accurately estimate the amount of claims that may be caused by various types of events and reasonably determine the insurance premium rate, and at the same time achieve a certain level of investment income on the basis of ensuring the safety and liquidity of funds to support the possible large-scale payment of claims. Finally, due to the diversification of business, after the outbreak of the epidemic, the real economy has suffered heavily, and the supply chain finance business has been seriously impacted, and various types of risks have come to the fore. Therefore, the multi-finance sector must adopt a diversified business layout and establish an extensive data collection system. This system should encompass not only the sector’s own business data, such as assets and liabilities, cash flow, and customers’ credit status, but also integrate external relevant data, including macroeconomic indicators, industry dynamics, and market fluctuation data. Furthermore, the sector should conduct regular stress tests for different types of emergencies to assess its risk exposure and implement appropriate measures in advance.

Secondly, for the government and regulatory authorities, it is necessary to improve the regulatory system of each financial subsector, reduce the dynamic risk spillover between sectors, and have the ability to prevent and control short-term risks under emergencies. Additionally, there is a need to strengthen the ability to measure and control long-term risks, establish a comprehensive and systematic financial risk prevention and control system, and prevent and mitigate financial systemic risks in advance.

In summary, this paper investigates the dynamic spillover effect situation of the risk of various financial sectors before, during and after the epidemic, which makes up for a gap in the research from this perspective. However, this paper exclusively focuses on the financial market and neglects to consider the influence of investors. Consequently, building on this paper, the dynamic spillover of investor sentiment on financial market risk can be further considered, which is the direction of further research by the authors, with a view to enabling better measurement of risk.

## Supporting information

S1 Dataset First. (CSV)

S2 Dataset Second. (CSV)

S3 Dataset Third. (CSV)

S4 Dataset Robustness check. (CSV)
